# Barleriside A, an aryl hydrocarbon receptor antagonist, ameliorates podocyte injury through inhibiting oxidative stress and inflammation

**DOI:** 10.3389/fphar.2024.1386604

**Published:** 2024-08-22

**Authors:** Xiao-Jun Li, Yan-Ni Wang, Wen-Feng Wang, Xiaoli Nie, Hua Miao, Ying-Yong Zhao

**Affiliations:** ^1^ School of Pharmacy, Zhejiang Chinese Medical University, Hangzhou, Zhejiang, China; ^2^ Department of Nephrology, Integrated Hospital of Traditional Chinese Medicine, Southern Medical University, Guangzhou, Guangdong, China; ^3^ School of Pharmacy, Heilongjiang University of Chinese Medicine, Harbin, Heilongjiang, China

**Keywords:** aryl hydrocarbon receptor, oxidative stress and inflammation, nuclear factor kappa B, Nrf2, chronic kidney disease, podocyte injury, *Plantaginis semen*, barleriside A

## Abstract

**Introduction:**

Increasing evidence shows that hyperactive aryl hydrocarbon receptor (AHR) signalling is involved in renal disease. However, no currently available intervention strategy is effective in halting disease progression by targeting the AHR signalling. Our previous study showed that barleriside A (BSA), a major component of *Plantaginis semen*, exhibits renoprotective effects.

**Methods:**

In this study, we determined the effects of BSA on AHR expression in 5/6 nephrectomized (NX) rats. We further determined the effect of BSA on AHR, nuclear factor kappa B (NF-ƙB), and the nuclear factor erythroid 2-related factor 2 (Nrf2) signalling cascade in zymosan-activated serum (ZAS)-stimulated MPC5 cells.

**Results:**

BSA treatment improved renal function and inhibited intrarenal nuclear AHR protein expression in NX-treated rats. BSA mitigated podocyte lesions and suppressed AHR mRNA and protein expression in ZAS-stimulated MPC5 cells. BSA inhibited inflammation by improving the NF-ƙB and Nrf2 pathways in ZAS-stimulated MPC5 cells. However, BSA did not markedly upregulate the expression of podocyte-specific proteins in the ZAS-mediated MPC5 cells treated with CH223191 or AHR siRNA compared to untreated ZAS-induced MPC5 cells. Similarly, the inhibitory effects of BSA on nuclear NF-ƙB p65, Nrf2, and AHR, as well as cytoplasmic cyclooxygenase-2, heme oxygenase-1, and AHR, were partially abolished in ZAS-induced MPC5 cells treated with CH223191 or AHRsiRNA compared with untreated ZAS-induced MPC5 cells. These results indicated that BSA attenuated the inflammatory response, partly by inhibiting AHR signalling.

**Discussion:**

Both pharmacological and siNRA findings suggested that BSA mitigated podocyte lesions by improving the NF-ƙB and Nrf2 pathways via inhibiting AHR signalling. Therefore, BSA is a high-affinity AHR antagonist that abolishes oxidative stress and inflammation.

## 1 Introduction

Aryl hydrocarbon receptor (AHR) is a cytoplasmic ligand-mediated transcription factor (Cao et al., 2022; Ouyang et al., 2020). The biological functions of AHR include immune regulation, cell cycle regulation, mucosal barrier function, and organogenesis, which are associated with ligand-mediated receptor activation (da Silva et al., 2022; Ouyang et al., 2020; Wu et al., 2022). AHR can transcribe various drug-metabolizing enzymes including *cytochrome P450 family 1 subfamily A member 1* (*CYP1A1*), *cytochrome P450 family 1 subfamily A member 2* (*CYP1A2*), and *cytochrome P450 family 1 subfamily B member 1* (*CYP1B1*) (Miao et al., 2020). High-affinity AHR ligands have been identified as xenobiotics, such as 2,3,7,8-tetrachlorodibenzo-p-dioxin (Cao et al., 2022). Previous study has suggested that hyperactive AHR signal was implicated in patients with podocyte damage-associated renal disease such as immunoglobulin A nephropathy (IgAN), diabetic kidney disease (DKD) and idiopathic membranous nephropathy (IMN) (Miao et al., 2020). Our previous study showed increased intrarenal AHR mRNA and protein expression in patients with chronic kidney disease (CKD) (Miao et al., 2022). However, no currently available therapy is effective for halting disease progression. Therefore, identification of novel AHR ligands plays a critical role in targeting this enigmatic receptor for the treatment of various diseases.

Mounting evidence suggests that traditional Chinese medicines (TCM) are a key source of new drugs and are used for treatment of various diseases (Newman and Cragg, 2020; Hou et al., 2022; Li, et al., 2022a; Marena et al., 2022; Zhao et al., 2022a; Kong et al., 2023). Natural compounds form TCM were widely used for improving renal function and treating renal injury (Ren et al., 2021; Hu et al., 2022; Zhou et al., 2022; Shao et al., 2023; Sun et al., 2023). Substantial achievements have shown a myriad of natural compounds that can directly modulate AHR signalling (Goya-Jorge et al., 2021). Our previous studies have demonstrated that a number of flavonoids, such as barleriside A (BSA), rhoifolin, 5,7,3′,4′,5′-pentahydroxy flavanone and 5,6,7,8,3′,4′-hexamethoxyflavone, and lignans, including matairesinol and erythro-guaiacylglycerol-β-ferulic acid ether as AHR antagonists, attenuate renal fibrosis by suppressing AHR signalling (Miao et al., 2020; Cao et al., 2022; Miao et al., 2022). In addition, astragaloside IV attenuates renal damage and AHR signalling in mice (Mo et al., 2023). *In vitro* experiments have shown that astragaloside IV suppresses inflammation and AHR signalling in indoxyl sulfate-treated HK-2 cells (Mo et al., 2023). Moreover, a previous study showed that lycopene mitigated Di (2-ethylhexyl) phthalate-induced renal cell injury by suppressing AHR signal (Li et al., 2021). Collectively, naturally derived compounds, such as AHR inhibitors, attenuated renal fibrosis.

Numerous studies have reported that inflammation plays a central role in CKD (Singh et al., 2022; Wang et al., 2023b; Yuan et al., 2022). *Cyclooxygenase-2* (*COX-2*) is produced by transcription factors including AHR and nuclear factor kappa B (NF-ƙB) p65. Several studies have shown that AHR interacts with NF-ƙB in CKD (Addi et al., 2019; Brito et al., 2019; Curran and Kopp, 2022). Our recent study showed that inhibitor of kappa B (IƙB)/NF-ƙB pathway was a downstream target of AHR signal in IMN (Wang et al., 2023b). However, there are no agents that inhibit NF-ƙB pathway by targeting AHR signalling. *Plantaginis semen* is widely used as a diuretic to improve renal function and treat renal diseases in patients (Wen et al., 2023a). Our previous study showed that BSA, a major component of *P. semen*, exhibits renoprotective effects (Miao et al., 2020). In this study, we first determined the effect of BSA on AHR signals in 5/6 nephrectomized (NX) rats. We further revealed that BSA, an AHR antagonist, ameliorated podocyte injury through IƙB/NF-ƙB and kelch-like ECH-associated protein 1 (Keap1)/nuclear factor erythroid 2-related factor 2 (Nrf2) signalling cascade in zymosan activation serum (ZAS)-stimulated MPC5 cells. Our study will uncover that BSA ameliorate podocyte damage-associated renal disease by improving IƙB/NF-ƙB and Keap1/Nrf2 pathways via suppressing hyperactive AHR expression.

## 2 Materials and methods

### 2.1 Chemicals, antibodies and reagents

Zymosan A was purchased from Sigma–Aldrich (St. Louis, MO, USA). Primary antibodies, Healthy human and Western quick horseradish peroxidase chemiluminescent substrate were presented in the previous publications (Wang et al., 2023).

### 2.2 NX-induced CKD rats treated by BSA

Male Sprague Dawley rats were purchased from the Animal Center of the Xi’an Jiaotong University (Xi’an, Shaanxi, China). NX rats were reproduced as described in the previous publication (Miao et al., 2020). Rats were divided into three groups: Sham, NX and NX + BSA (n = 8/group). BSA was administered at 10 mg/kg/day by gastric irrigation for 4 weeks from ninth to 12th week. Urine was collected for 24 h after 12 weeks. All rats were euthanized after anesthetization with 10% urethane. Serum and kidney tissue samples were also collected. The animal care and experiments are approved by Ethics Committee for Animal Experiments of University (No. 20200713-06).

### 2.3 ZAS preparation and cell treatment

Mouse podocyte cell culture was performed as described in the previous publication (Wang et al., 2023a). C5b-9 was prepared as described in our previous study (Wang et al., 2023a). MPC5 cells were stimulated with 10% ZAS for 24 h in the absence or presence of BSA (20 μM) and CH223191 (10 μM). The treated cells were collected.

### 2.4 Serum and urine biochemical analysis

Creatinine and urea levels in serum were measured using a Beckman AU680 automatic analyzer. Proteinuria was measured using a Roche Cobas C501 Chemistry Analyzer.

### 2.5 Quantitative real-time polymerase chain reaction (RT-PCR)

Extracted total RNA, quantitative RT-PCR and specific primers were shown in the previous publications (Miao et al., 2020; Cao et al., 2022; Miao et al., 2022).

### 2.6 Immunohistochemistry

Kidneys were incubated overnight at 4°C with an anti-AHR primary antibody, and then incubated with a secondary antibody. Analysis was carried out using a light microscope.

### 2.7 Immunofluorescence

MPC5 cells were incubated with antibodies against podocin, AHR, NF-ƙB p65, COX-2, Nrf2, and haem oxygenase-1 (HO-1). Details of the immunofluorescence methods are presented in the previous publication (Wang et al., 2023).

### 2.8 Western blot analysis

Western blot analysis was performed in the previous publication (Cao et al., 2022). The expression levels were normalized to those of α-tubulin and histone H3. The relative levels were quantified using the ImageJ software.

### 2.9 Statistical analysis

The experimental results are presented as the mean ± standard error of mean. The statistical significance was analyzed using one-way ANOVA using the GraphPad Prism software. The values for *P* < 0.05 were considered statistically significant.

## 3 Results

### 3.1 BSA improved kidney function and suppressed nuclei AHR expression in NX-induced CKD rats

**FIGURE 1:**
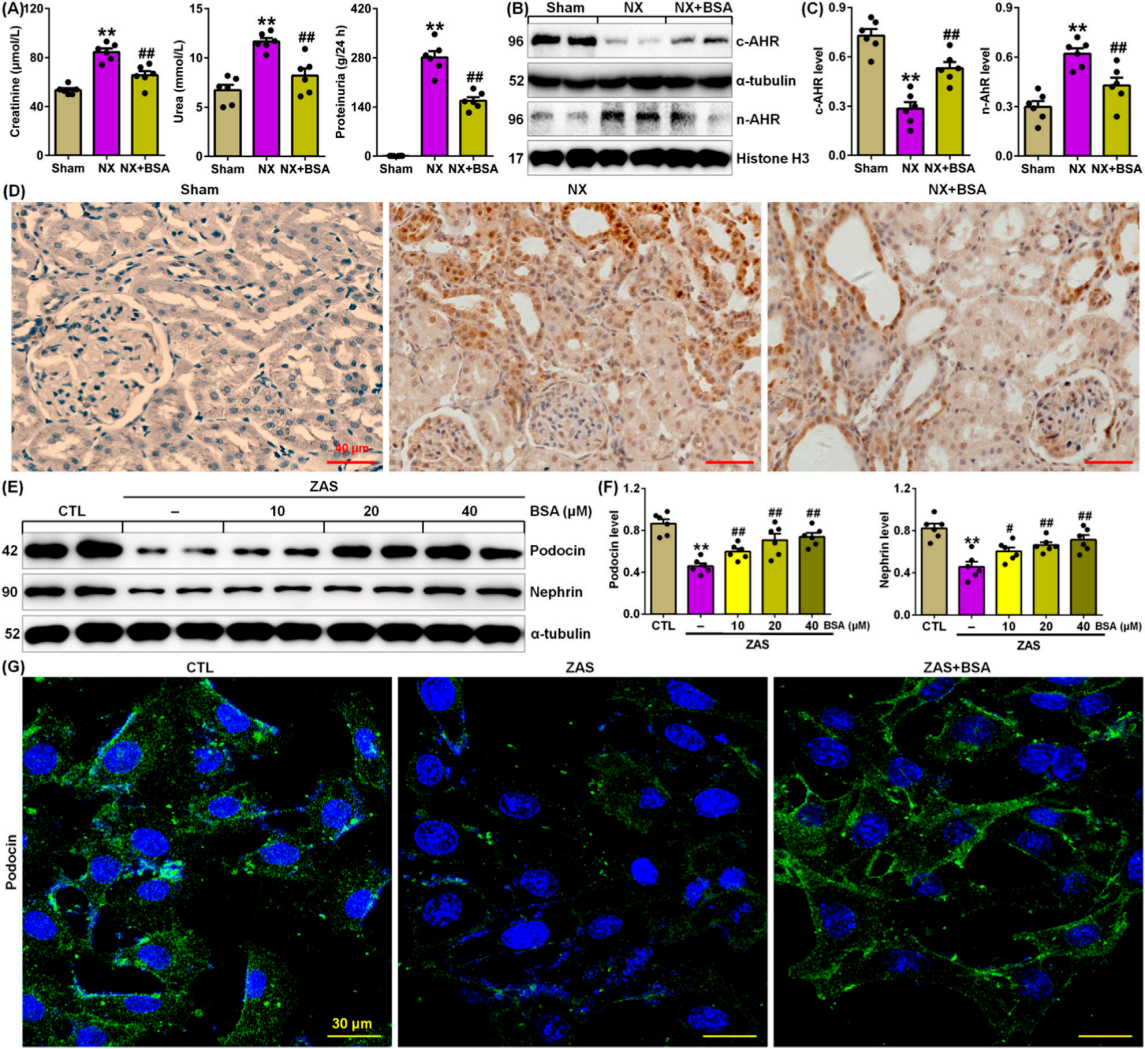
BSA inhibits AHR expression in the NX-induced rats. **(A)** Serum levels of creatinine and urea and proteinuria in the control and NX-induced rats with or without BSA. **(B)** Cytoplasm and nuclei AHR protein expression in the renal tissues of control and NX-induced rats with or without BSA. **(C)** Protein levels of cytoplasm and nuclei AHR in the renal tissues of control and NX-induced rats with or without BSA. **(D)** Immunohistochemical analysis with intrarenal anti-AHR antibody in the control and NX-induced rats with or without BSA. **(E)** Protein expression levels of podocin and nephrin in the ZAS-induced MPC5 cells treated with the different concentrations of BSA. **(F)** Quantitative analysis of protein expression of podocin and nephrin in ZAS-induced MPC5 cells treated with the different concentrations of BSA. **(G)** Immunofluorescent analysis with anti-podocin antibody in the control and ZAS-stimulated MPC5 cells with or without BSA. ^*^ *P* < 0.05, ^**^ *P* < 0.01 compared with sham or CTL; ^#^ *P* < 0.05, ^##^ *P* < 0.01 compared with NX or ZAS-stimulated MPC5 cells.

Compared to Sham rats, NX rats presented a markedly increase in the serum levels of creatinine and urea, as well as proteinuria levels, while BSA treatment markedly reduced levels of three renal function markers in NX-induced CKD rats (Figure 1A), indicating that BSA improved renal function in NX-induced CKD rats.

Compared to Sham rats, NX rats presented a significant reduction in intrarenal cytoplasmic AHR protein expression in NX-induced CKD rats, which was accompanied by a significant increase in intrarenal nuclei AHR protein expression in NX-induced CKD rats (Figures 1B, C). BSA treatment markedly preserved cytoplasmic AHR protein expression and markedly reduced nuclear AHR protein expression in renal tissues of NX-induced CKD rats (Figures 1B, C). This result was further verified using immunohistochemical analysis (Figure 1D). These data suggest that activating AHR signalling in CKD and AHR may be an effective therapeutic target. BSA is a novel aryl hydrocarbon receptor antagonist.

To elucidate the renoprotective mechanism of BSA, we first determined its effect on ZAS-stimulated MPC5 cells. BSA could markedly inhibit the downregulation of the protein expression of podocin and nephrin in ZAS-treated MPC5 cells in a concentration-dependent manner (10–40 μM) within 24 h (Figures 1E, F). The concentrations of 20 and 40 μM BSA had a stronger effect on the upregulated podocin and nephrin protein expressions than did 10 μM BSA (Figures 1E, F). But, the effect at 20 μM was similar to that observed at 40 μM. Therefore, 20 μM was used for this experiment. Immunofluorescence staining further verified that BSA treatment preserved podocin expression in the ZAS-stimulated MPC5 cells (Figure 1G).

### 3.2 BSA inhibited AHR signalling in the ZAS-stimulated MPC5 cells

**FIGURE 2:**
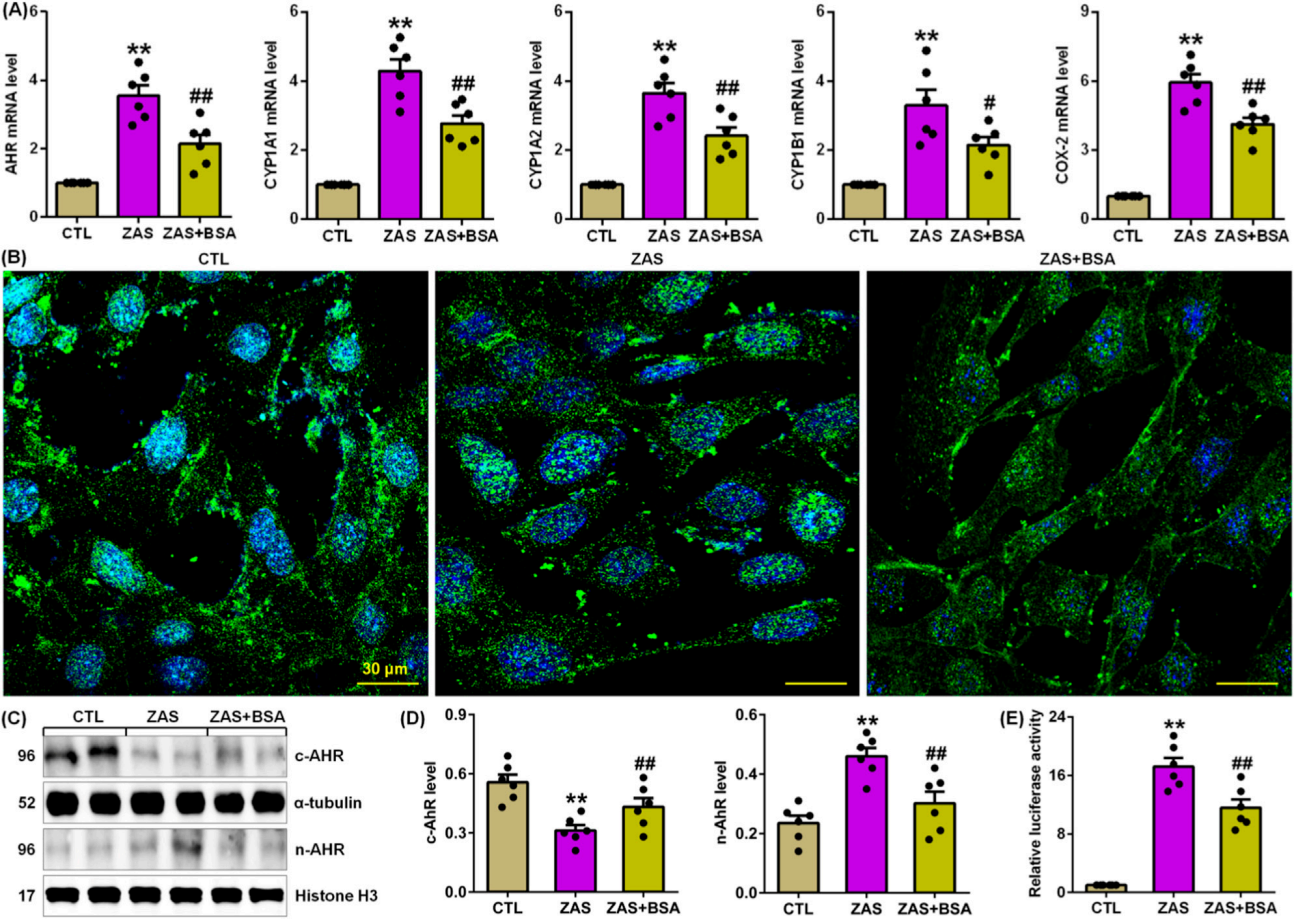
BSA inhibits AHR signalling in the ZAS-stimulated MPC5 cells. **(A)** The mRNA levels of *AHR* and its target genes, including *CYP1A1*, *CYP1A2*, *CYP1B1* and *COX-2* in the control and ZAS-stimulated MPC5 cells with or without BSA. **(B)** Immunofluorescent analysis with anti-AHR antibody in the control and ZAS-stimulated MPC5 cells with or without BSA. **(C)** Cytoplasm and nuclei AHR protein expression in the control and ZAS-stimulated MPC5 cells with or without BSA. **(D)** Protein levels of cytoplasm and nuclei AHR in the control and CBSA-induced MN rats with or without MSG. **(E)** Luciferase assay of AHR activation in the control and ZAS-stimulated MPC5 cells with or without BSA. ^*^ *P* < 0.05, ^**^ *P* < 0.01 compared with CTL; ^#^ *P* < 0.05, ^##^ *P* < 0.01 compared with ZAS-stimulated MPC5 cells.

Compared to ZAS-stimulated MPC5 cells, BSA treatment markedly inhibited the mRNA expression of *AHR* and its four target genes, such as *CYP1A1*, *CYP1A2*, *CYP1B1* and *COX-2* in ZAS-induced MPC5 cells (Figure 2A), which was accompanied by reduced AHR nuclear translocation (Figure 2B), which was in line with the protein expression of increasing cytoplasmic AHR and decreasing nuclear AHR (Figures 2C, D). Luciferase assay uncovered that BSA treatment markedly inhibited AHR-driven reporter activity in ZAS-stimulated MPC5 cells (Figure 2E). These data indicate that BSA inhibits activating AHR signalling in ZAS-stimulated MPC5 cells.

### 3.3 BSA inhibited hyperactive IƙB/NF-ƙB pathway in the ZAS-stimulated MPC5 cells

**FIGURE 3:**
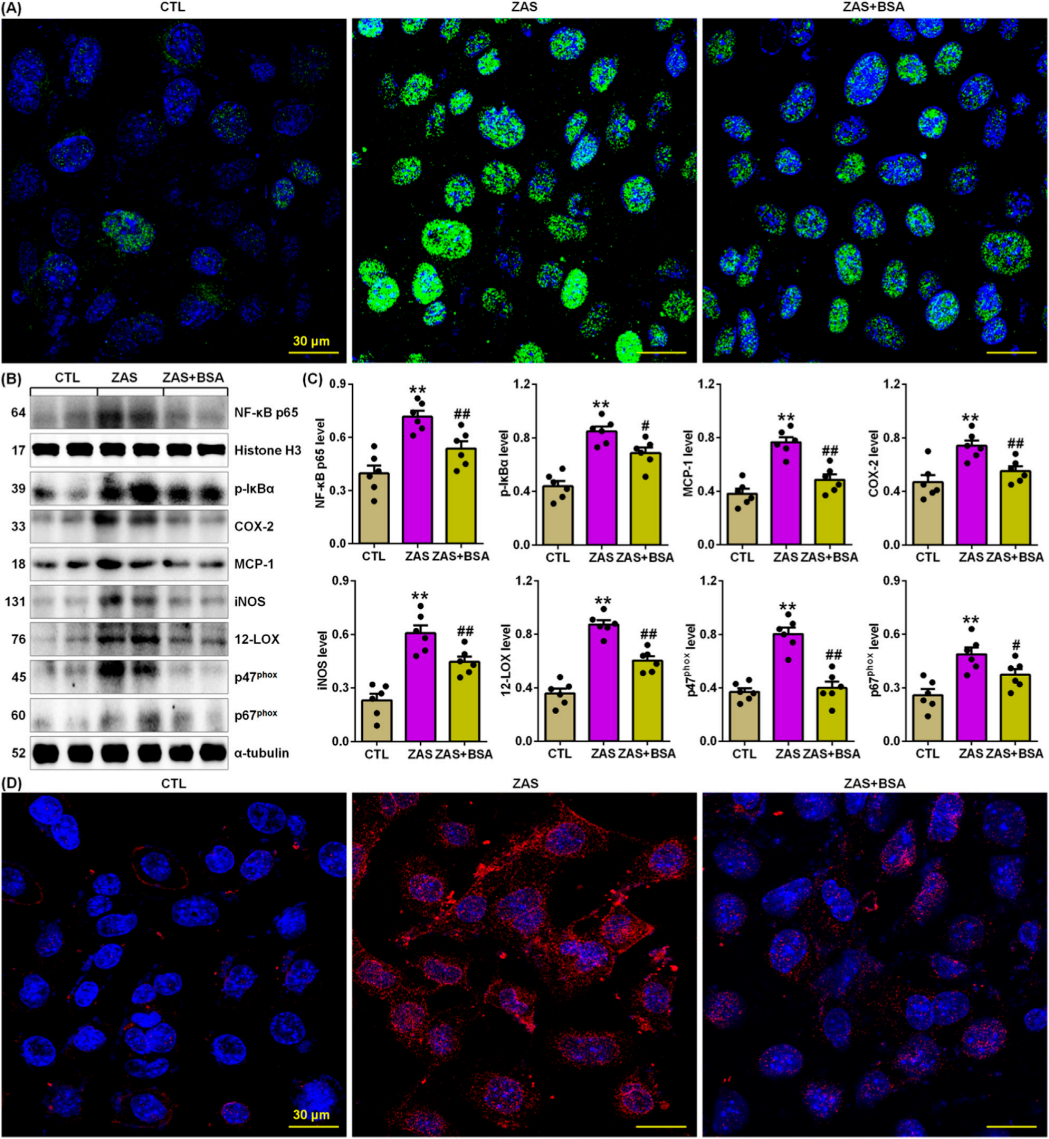
BSA inhibits activation of IƙB/NF-ƙB pathway in the ZAS-stimulated MPC5 cells. **(A)** Immunofluorescence analysis with anti-p65 antibody in the control and ZAS-stimulated MPC5 cells with or without BSA. **(B)** Protein expression of p-IƙBα and nuclei p65 and its downstream gene products including COX-2, MCP-1, iNOS, 12-LOX, p47^phox^ and p67^phox^ in the control and ZAS-stimulated MPC5 cells with or without BSA. **(C)** Protein levels of p-IƙBα, NF-ƙB p65, COX-2, MCP-1, iNOS, 12-LOX, p47^phox^ and p67^phox^ in the control and ZAS-stimulated MPC5 cells with or without BSA. **(D)** Immunofluorescence analysis with and COX-2 antibody in the control and ZAS-stimulated MPC5 cells with or without BSA. ^*^ *P* < 0.05, ^**^ *P* < 0.01 compared with CTL; ^#^ *P* < 0.05, ^##^ *P* < 0.01 compared with ZAS-stimulated MPC5 cells.

Compared to ZAS-stimulated MPC5 cells, BSA treatment markedly reduced nuclear p65 expression in ZAS-stimulated MPC5 cells (Figures 3A–C). This is acspanied by markedly inhibiting protein expressions of p-IƙBα and p65 downstream target gene products such as COX-2, inducible nitric oxide synthase (iNOS), monocyte chemotactic protein-1 (MCP-1), 12-lipoxygenase (12-LOX), p67^phox^ and p67^phox^ in the ZAS-stimulated MPC5 cells (Figures 3B, C), which were consistent with markedly inhibiting cytoplasm COX-2 protein expression in the ZAS-stimulated MPC5 cells (Figure 3D). Therefore, these data suggest that BSA inhibits the hyperactive IƙB/NF-ƙB pathway in ZAS-stimulated MPC5 cells.

### 3.4 BSA improved impaired Keap1/Nrf2 pathway in the ZAS-stimulated MPC5 cells

**FIGURE 4:**
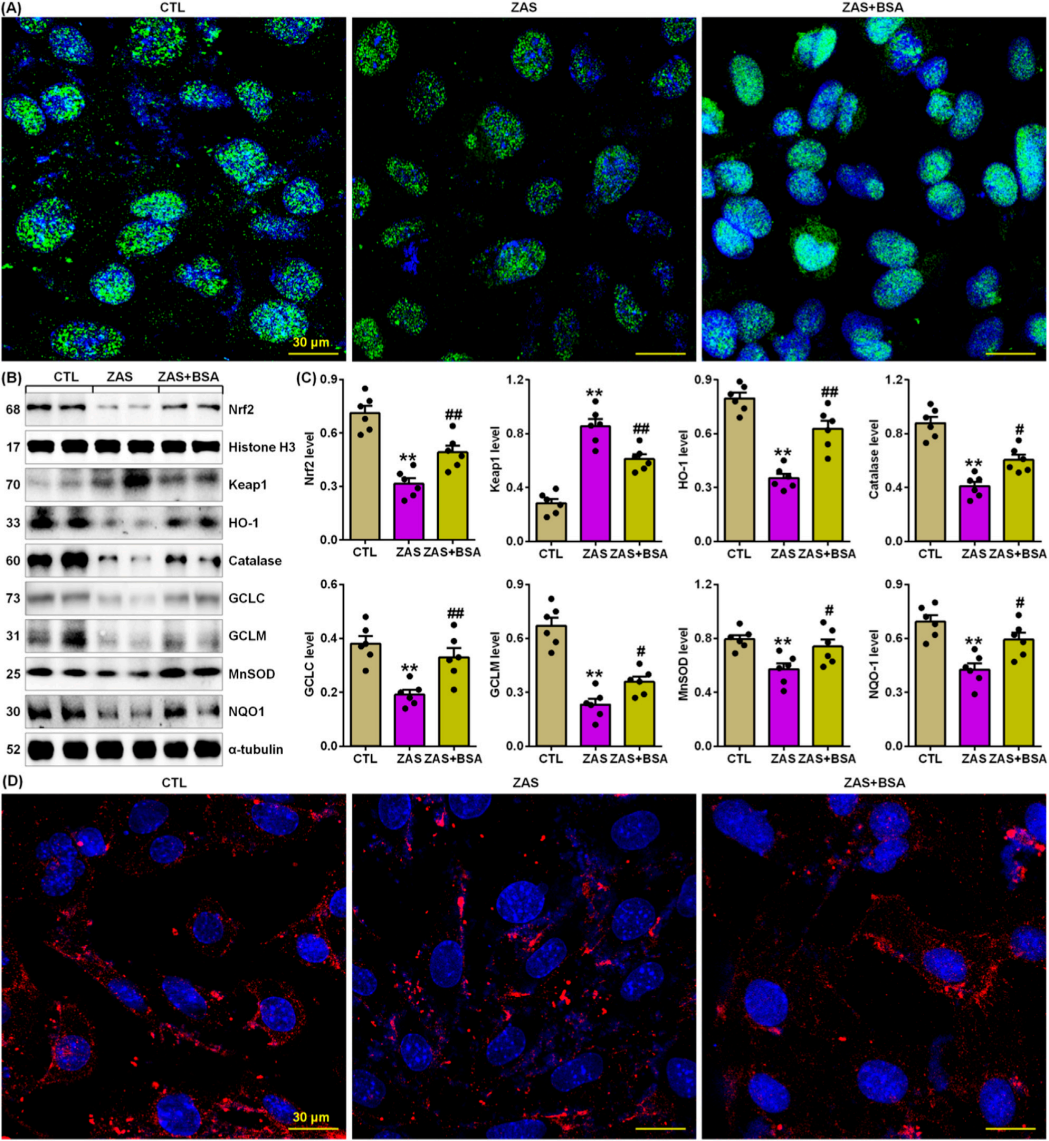
BSA improves impairment of Keap1/Nrf2 pathway in the ZAS-stimulated MPC5 cells. **(A)** Immunofluorescence analysis with anti-Nrf2 antibody in the control and ZAS-stimulated MPC5 cells with or without BSA. **(B)** Protein expression of Nrf2, Keap1, HO-1, catalase, GCLC, GCLM, MnSOD and NQO-1 in the control and ZAS-stimulated MPC5 cells with or without BSA. **(C)** Protein levels of Nrf2, Keap1, HO-1, catalase, GCLC, GCLM, MnSOD and NQO-1 in the control and ZAS-stimulated MPC5 cells with or without BSA. **(D)** Immunofluorescence analysis with anti-HO-1 antibody in the control and ZAS-stimulated MPC5 cells with or without BSA. ^*^ *P* < 0.05, ^**^ *P* < 0.01 compared with CTL; ^#^ *P* < 0.05, ^##^ *P* < 0.01 compared with ZAS-stimulated MPC5 cells.

Compared to untreated ZAS-stimulated MPC5 cells, treatment with BSA markedly increased nuclear Nrf2 expression in ZAS-stimulated MPC5 cells (Figures 4A–C). This is also accompanied by markedly reduced protein expression of Keap1 and increased Nrf2 target gene products, such as HO-1, catalase, glutamate-cysteine ligase catalytic subunit (GCLC), glutamate-cysteine ligase modifier subunit (GCLM), manganese superoxide dismutase (MnSOD), and nicotinamide adenine dinucleotide phosphate quinone dehydrogenase 1 (NQO-1), in ZAS-stimulated MPC5 cells treated with BSA compared to untreated ZAS-stimulated MPC5 cells (Figures 4B, C). In addition, BSA treatment markedly increased cytoplasmic HO-1 protein expression in ZAS-stimulated MPC5 cells (Figure 4D). These results suggest that BSA improves the impaired Keap1/Nrf2 pathway in ZAS-induced MPC5 cells. Collectively, these results indicate that BSA improves the activating IƙB/NF-ƙB and impaired Keap1/Nrf2 pathway in MPC5 cells.

### 3.5 BSA ameliorated podocyte injury by improving IƙB/NF-ƙB and Keap1/Nrf2 pathways via AHR signal

**FIGURE 5:**
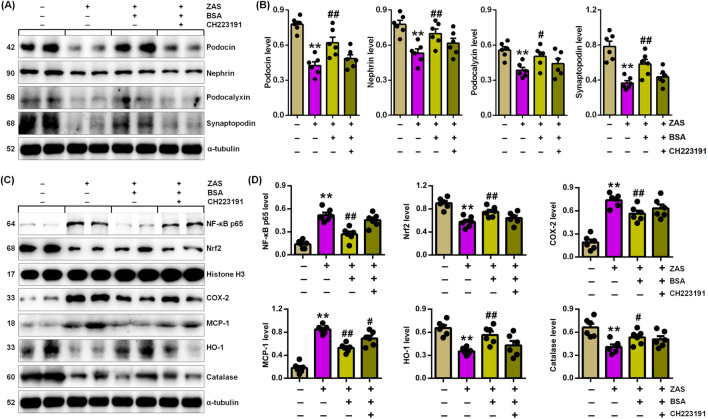
BSA inhibits podocyte injury through improving IƙB/NF-ƙB and Keap1/Nrf2 pathways via AHR signalling in the ZAS-induced MPC5 cells with BSA and/or CH223191. **(A)** Podocyte-specific protein expression in the ZAS-induced MPC5 cells with BSA and/or CH223191. **(B)** Podocyte-specific protein levels in the ZAS-induced MPC5 cells with BSA and/or CH223191. **(C)** Protein expression of NF-ƙB p65, Nrf2, COX-2, MCP-1, HO-1 and catalase in the ZAS-induced MPC5 cells with BSA and/or CH223191. **(D)** Protein levels of NF-κB p65, Nrf2, COX-2, MCP-1, HO-1 and catalase in the ZAS-induced MPC5 cells with BSA and/or CH223191. ^*^ *P* < 0.05, ^**^ *P* < 0.01 compared with CTL; ^#^ *P* < 0.05, ^##^ *P* < 0.01 compared with ZAS-stimulated MPC5 cells.

**FIGURE 6:**
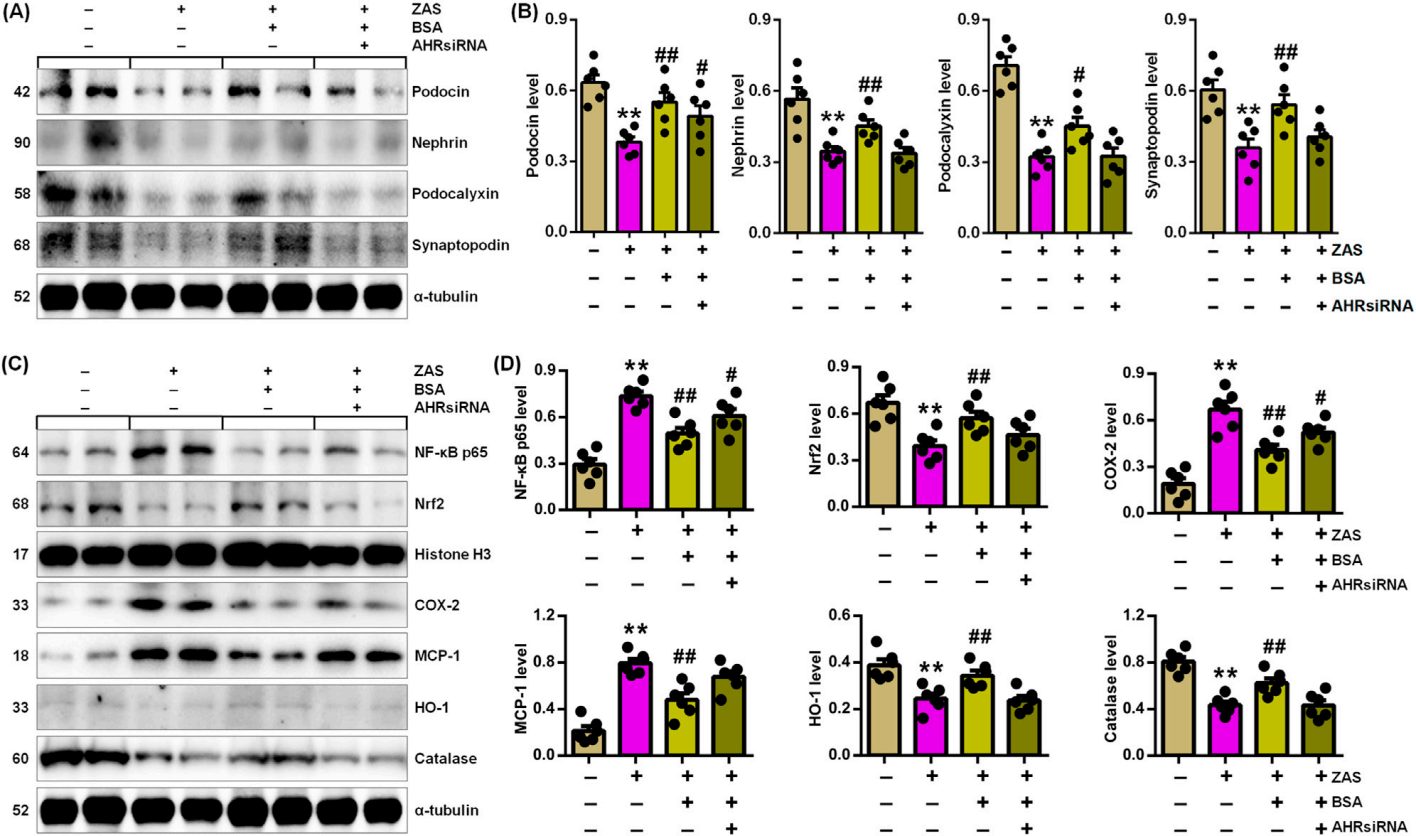
BSA inhibits podocyte injury through improving IƙB/NF-ƙB and Keap1/Nrf2 pathways via AHR signalling in the ZAS-induced AHRsiRNA-transfected MPC5 cells treated with BSA. **(A)** Podocyte-specific protein expression in the ZAS-induced AHRsiRNA-transfected MPC5 cells treated with BSA. **(B)** Podocyte-specific protein levels in the ZAS-induced AHRsiRNA-transfected MPC5 cells treated with BSA. **(C)** Protein expression of NF-ƙB p65, Nrf2, COX-2, MCP-1, HO-1 and catalase in the ZAS-induced AHRsiRNA-transfected MPC5 cells treated with BSA. **(D)** Protein levels of NF-ƙB p65, Nrf2, COX-2, MCP-1, HO-1 and catalase in the ZAS-induced AHRsiRNA-transfected MPC5 cells treated with BSA. ^*^ *P* < 0.05, ^**^ *P* < 0.01 compared with CTL; ^#^ *P* < 0.05, ^##^ *P* < 0.01 compared with ZAS-stimulated MPC5 cells.

We determine whether BSA mitigates MPC5 cell damage by improving IƙB/NF-ƙB and Keap1/Nrf2 pathways via suppressing AHR signalling. BSA treatment downregulated the expression of podocyte-specific proteins in ZAS-mediated MPC5 cells (Figures 5A, B). However, BSA did not significantly upregulate podocyte protein expression in ZAS-induced MPC5 cells treated with CH223191 compared with only ZAS-mediated MPC5 cells (Figures 5A, B). Similarly, the inhibitory effect of BSA on nuclear NF-ƙB p65 and Nrf2 as well as cytoplasmic COX-2, MCP-1, HO-1 and catalase was partially abolished in ZAS-mediated MPC5 cells treated with CH223191 compared with only ZAS-induced MPC5 cells (Figures 5C, D). The results were also demonstrated in the ZAS-induced AHR siRNA-transfected MPC5 cells treated with BSA (Figures 6A, D). These results indicate that BSA attenuates inflammatory response via inhibiting AHR signalling. Totally, both pharmacological and siRNA results demonstrate that BSA attenuates podocyte lesion by modulating IƙB/NF-ƙB and Keap1/Nrf2 pathways via suppressing AHR signalling.

## 4 Discussion

Increasing publications have suggested that TCM improved various diseases by regulating AHR signalling (Wen et al., 2023b; Ying et al., 2024; Zhang et al., 2023; Wang et al., 2024). Accumulated evidence has showed increasing serum AHR activity in CKD patients (Dou et al., 2018; Kim et al., 2013; Kim et al., 2020). Dou et al. demonstrated that CKD patients with stages 3–5 showed strong serum AHR-activating potential and upregulated mRNA levels of *CYP1A1* and *AHR repressor* in whole blood compared to serum from healthy controls (Dou et al., 2018). Kim et al. demonstrated that serum AHR transactivation activity was higher in DKD patients with microalbuminuria and macroalbuminuria than in those with normoalbuminuria (Kim et al. (2013), indicating that high serum AHR transactivation is a high risk factor for DKD. The same research group further demonstrated that serum AHR transactivation activity was increased in non-dialysis CKD patients compared to patients on dialysis, whereas its activity was increased in patients undergoing hemodialysis compared to undergoing peritoneal dialysis (Kim et al., 2020). Hemodialysis treatment could decrease AHR transactivation activity in patients with hemodialysis dialysis (Kim et al., 2020). Some studies have shown hyperactive AHR signalling in renal tissues of CKD patients and animal models (Miao et al., 2020; Miao et al., 2022; Cao et al., 2022; Miao et al., 2024). First, our previous study demonstrated increased intrarenal mRNA expression of *AHR* and its genes, such as *CYP1A1*, *CYP1A2* and *CYP1B1* in CKD patients at five stages, accompanied by elevating AHR nuclear translocation (Miao et al., 2022). Second, our previous study revealed elevated intrarenal AHR nuclear translocation in patients with DKD, IgAN and IMN (Miao et al., 2020). Our latest study further showed increased intrarenal mRNA expression of *AHR* and its genes, including *CYP1A1*, *CYP1A2* and *CYP1B1* in patients with IMN, accompanied by elevated AHR nuclear translocation (Miao et al., 2024; Wang et al., 2023). Similar findings were also demonstrated in several rat or mice models treated with NX, adenine, unilateral ureteral obstruction and cationic bovine serum albumin (CBSA) (Miao et al., 2020; Cao et al., 2022; Miao et al., 2022). These data show that AHR signalling is activated in various pathological types of CKD. Therefore, AHR is a promising therapeutic target for improving renal function in CKD patients.

**FIGURE 7:**
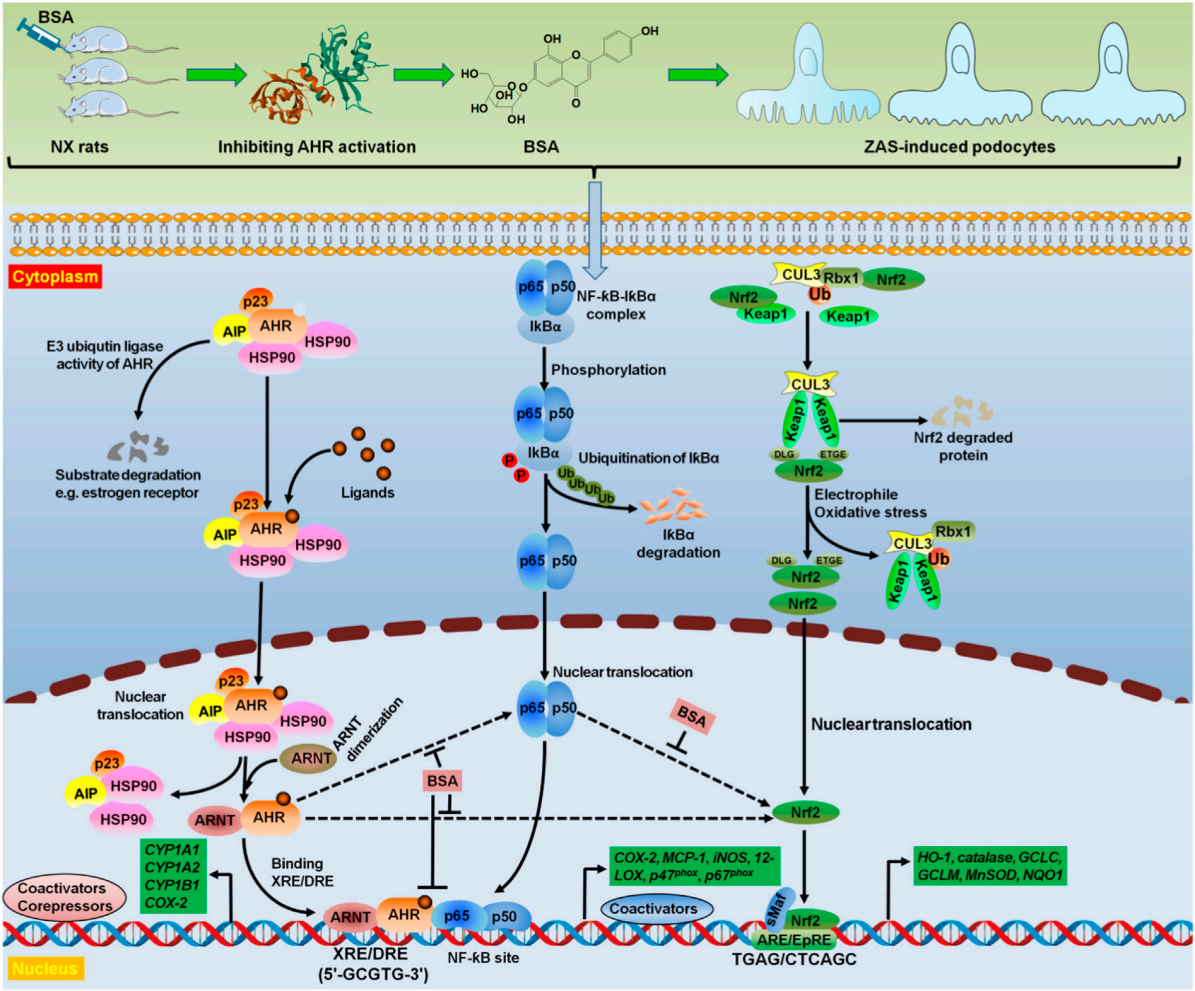
BSA as a novel AHR antagonist targeted oxidative stress and inflammation. Treatment with BSA suppressed intrarenal AHR expression at both mRNA and protein levels in NX rats. Hyperactive AHR and IƙB/NF-ƙB pathways and impaired Keap1/Nrf2 pathway were demonstrated in the ZAS-stimulated MPC5 cells. However, treatment with BSA could suppress hyperactive AHR and IƙB/NF-ƙB pathways and enhance impaired Keap1/Nrf2 pathway in the ZAS-stimulated MPC5 cells. Mechanistically, both pharmacological and genetic methods suggested that BSA ameliorated podocyte injury by improving IƙB/NF-ƙB and Keap1/Nrf2 pathways via AHR signalling. Therefore, BSA suppressed intrarenal AHR expression at both mRNA and protein levels by using *in vivo* and *in vitro* experiments. BSA was demonstrated to be as a high-affinity AHR antagonist that abolished oxidative stress and inflammation. AIP, AHR interacting protein; ARE, antioxidant response element; ARE/EpRE antioxidant/electrophile response element; ARNT, aryl hydrocarbon receptor nuclear translocator; CUL3, Cullin3; HSP90, heat shock protein 90; Rbx1, ring-Box 1; sMaf, small musculoaponeurotic fibrosarcoma; Ub, ubiquitin.

Natural products have been demonstrated to be effective therapies for intervention in glomerular-related diseases including glomerulonephritis (Wang et al., 2021; Zhao et al., 2022b; Qin et al., 2023), DKD (Huang et al., 2022; Li et al., 2022b; Liu et al., 2022b; Pei et al., 2022) and IMN (Miao et al., 2024; Wang et al., 2023). In this study, we identified BSA as an AHR antagonist and it could ameliorate podocyte lesion through improving IƙB/NF-ƙB and Keap1/Nrf2 pathways (Figure 7). BSA inhibited the mRNA expression of *AHR*, *CYP1A1*, *CYP1A2*, *CYP1B1* and *COX-2* in ZAS-stimulated MPC5 cells, which was accompanied by inhibiting nuclear translocation of AHR. Accumulating evidence suggests that many natural product-derived components can directly regulate AHR signalling. Previous studies have shown that AHR ligands from vegetable extracts mediate *CYP1A1* activity (Zhao et al., 2019). Cruciferous family members, including broccoli, cauliflower, white cabbage, and Brussels sprouts, contain rich sources of AHR ligands, such as indole-3-carbinol and indole-3-acetonitrile (Zhao et al., 2019). Polyphenols are common components of the plant kingdom. Polyphenols are divided into five categories according to their chemical structures: phenolic acids, flavonoids, lignans, tannins and stilbenes. Extensive studies have demonstrated that phenolic acids and flavonoids are the most affluent polyphenolic components in diet and can be classified into flavanols, flavonols, flavones, flavanones, isoflavones, anthocyanins and proanthocyanidins (Zhao et al., 2019).

Recent studies suggested that TCM including Bupi Yishen formula, Dahuang Fuzi decoction and Jian-Pi-Yi-Shen formula attenuated CKD by inhibiting AHR signalling (Mo et al., 2021; Gu et al., 2022; Liu et al., 2022a). Our previous publications have demonstrated that some compounds such as matairesinol, rhoifolin, 5,6,7,8,3′,4′-hexamethoxyflavone, 5,7,3′,4′,5′-pentahydroxy flavanone and erythro-guaiacylglycerol-β-ferulic acid ether attenuated renal fibrosis by suppressing AHR signalling (Miao et al., 2020; Cao et al., 2022; Miao et al., 2022). BSA is a flavonoid glycoside. Previous studies suggested that BSA could decrease the activities of superoxide scavenging and xanthine oxidase, as well as inhibit the protein expression of extracellular matrix proteins, including collagen I, α-smooth muscle actin, and fibronectin in NRK-52E cells mediated by 1-aminopyrene (Karim et al., 2009; Miao et al., 2020). Our previous study showed that BSA inhibited mRNA expression of *AHR*, *CYP1A1*, *CYP1A2* and *CYP1B1* in renal tissues of NX-induced rats and NRK-52E cells mediated by 1-aminopyrene, which was accompanied by the protein expression of upregulated cytoplasmic AHR and downregulated nuclear AHR (Miao et al., 2020). Molecular ligand docking analysis revealed that BSA could bind to the active AHR site and exhibited a strong interaction with AHR. Collectively, the current study and other studies suggest that BSA is an effective AHR antagonist and suppresses AHR expression using *in vivo* and *in vitro* experiments (Figure 7).

Mechanistically, this study further illuminated that treatment with BSA mitigated podocyte lesion by suppressing hyperactive IƙB/NF-ƙB pathway and enhancing impaired Keap1/Nrf2 pathway via inhibiting AHR signalling in the ZAS-stimulated MPC5 cells (Figure 7). Both oxidative stress and inflammation change expression of a number of genes, including *NF-ƙB* and *Nrf2*. Our latest study showed that the NF-ƙB signalling was a downstream target of AHR pathway in IMN (Wang et al., 2023). Several studies have demonstrated that AHR interacts with NF-ƙB in CKD (Addi et al., 2019; Brito et al., 2019; Curran and Kopp, 2022). Brito et al. reported that increasing AHR protein levels were positively associated with increasing NF-ƙB protein levels in hemodialysis and non-dialysis-dependent patients (Brito et al., 2019). Our latest study showed increased protein expression of nuclear AHR and cytoplasmic COX-2 in the renal tissues of IMN patients (Wang et al., 2023). In addition, increased protein expression of nuclear AHR and cytoplasmic COX-2 was observed in renal tissues of rats treated with CBSA and ZAS-mediated MPC5 cells (Ma et al., 2023; Wang et al., 2023). Treatment with Moshen granules inhibited their expression in the renal tissues of rats treated with CBSA (Ma et al., 2023). Addi et al. demonstrated that an AHR ligand indole-3 acetic acid mediated activating tissue factor via AHR/NF-ƙB pathway (Addi et al., 2019). This research group further revealed that COX-2 levels were markedly suppressed in indole-3 acetic acid-induced umbilical vein endothelial cells treated with BAY 11-7082 and CH223191 (Dou et al., 2015). In addition, Lee et al. reported that ochratoxin A-treated HK-2 cells showed increased mRNA expression of *AHR* and its target genes, such as *CYP1A1* and *CYP1A2* representing phase I enzymes, as well as upregulated mRNA expression of phase II enzymes, such as *GCLC*, *NQO1* and *HO-1* by the activation of Nrf2 translocation (Lee et al., 2018). However, AHR deficiency ameliorates oxidative stress-induced macrophage infiltration, activating mesangial cell and kidney fibrosis in DKD mice (Lee et al., 2016).

Accumulated evidence has reported that renoprotective effect of natural products were associated with suppressing AHR, IƙB/NF-ƙB and Keap1/Nrf2 pathways. Dhulkifle et al. reported that treatment with 6-formylindolo(3,2-b)carbazole improved septic acute kidney injury and inflammation by increasing intrarenal mRNA expression of *H O -1* and *NQO1* via *AHR* and *Nrf2* (Dhulkifle et al., 2023). Recent publication showed that Dahuang Fuzi decoction blunted CKD by suppressing AHR/NF-ƙB pathway (Gu et al., 2022). Moreover, Zhao et al. showed that the beneficial effect of Tangshen formula for NF-ƙB p-p65 expression was related to AHR inhibition in renal tissues of DKD rats (Zhao et al., 2020). Our earlier publication showed that poricoic acids abolished AHR, IƙB/NF-ƙB and Keap1/Nrf2 pathways in mice with renal fibrosis (Wang et al., 2020). Collectively, this study demonstrated that BSA dampened podocyte lesion partly by modulating IƙB/NF-ƙB and Keap1/Nrf2 pathways via attenuating AHR signalling.

## 5 Conclusion

In conclusion, this study showed that treatment with BSA suppressed AHR expression at both the mRNA and protein levels in the renal tissues of NX rats and ZAS-stimulated MPC5 cells. We further illuminated that BSA mitigated podocyte lesion by suppressing hyperactive IƙB/NF-ƙB pathway and enhancing hypoactive Keap1/Nrf2 pathway via inhibiting AHR signalling in the ZAS-stimulated MPC5 cells. Mechanistically, both pharmacological and genetic results suggested that BSA ameliorated podocyte damage by modulating IƙB/NF-ƙB and Keap1/Nrf2 pathways via AHR signalling. Therefore, BSA is a high-affinity AHR antagonist that abolishes oxidative stress and inflammation. These findings may provide a leading drug for treating podocyte damage-related renal disease through oxidative stress and inflammation.

## Data Availability

The original contributions presented in the study are included in the article/Supplementary Material, further inquiries can be directed to the corresponding authors.
