# Supplementary figures and images for: Barleriside A, an aryl hydrocarbon receptor antagonist, ameliorates podocyte injury through inhibiting oxidative stress and inflammation
Source: Front Pharmacol. 2024 Aug 22;15:1386604. doi: 10.3389/fphar.2024.1386604 (PMC11374728; doi:10.3389/fphar.2024.1386604)

Figure 1B

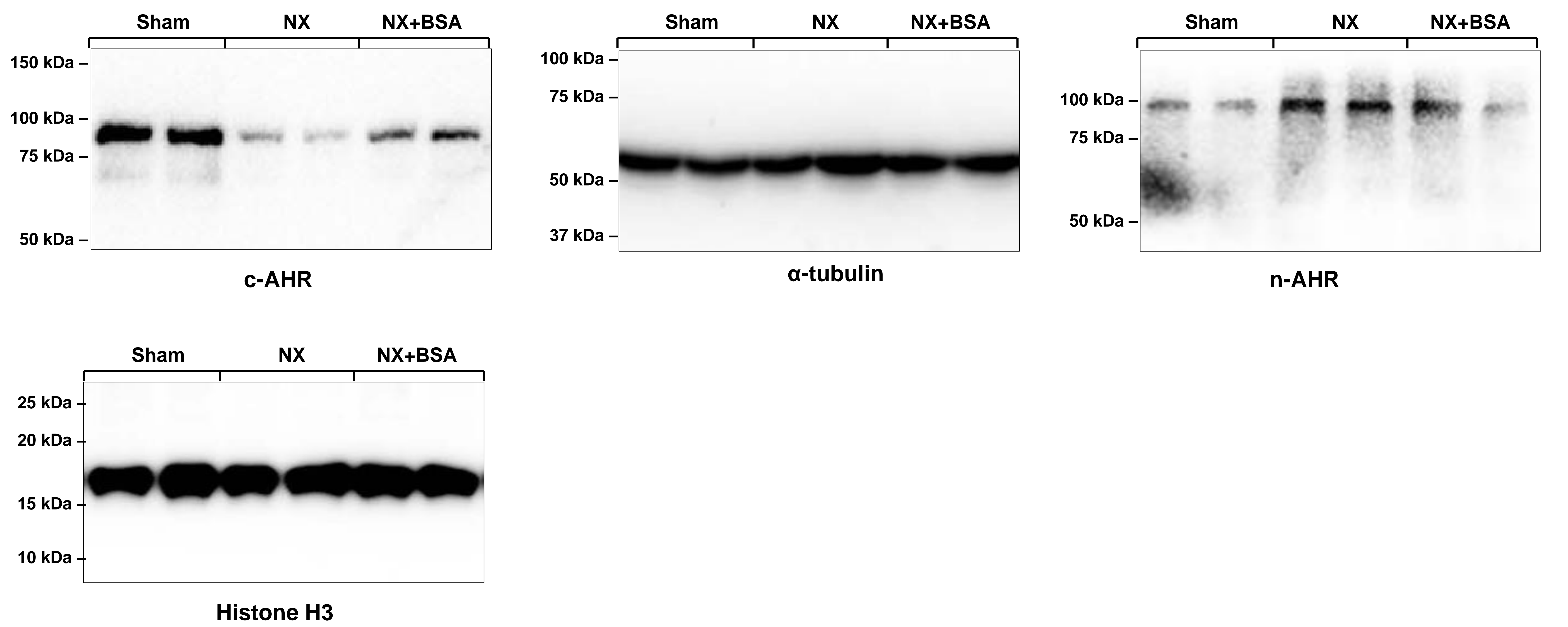

Figure 1E

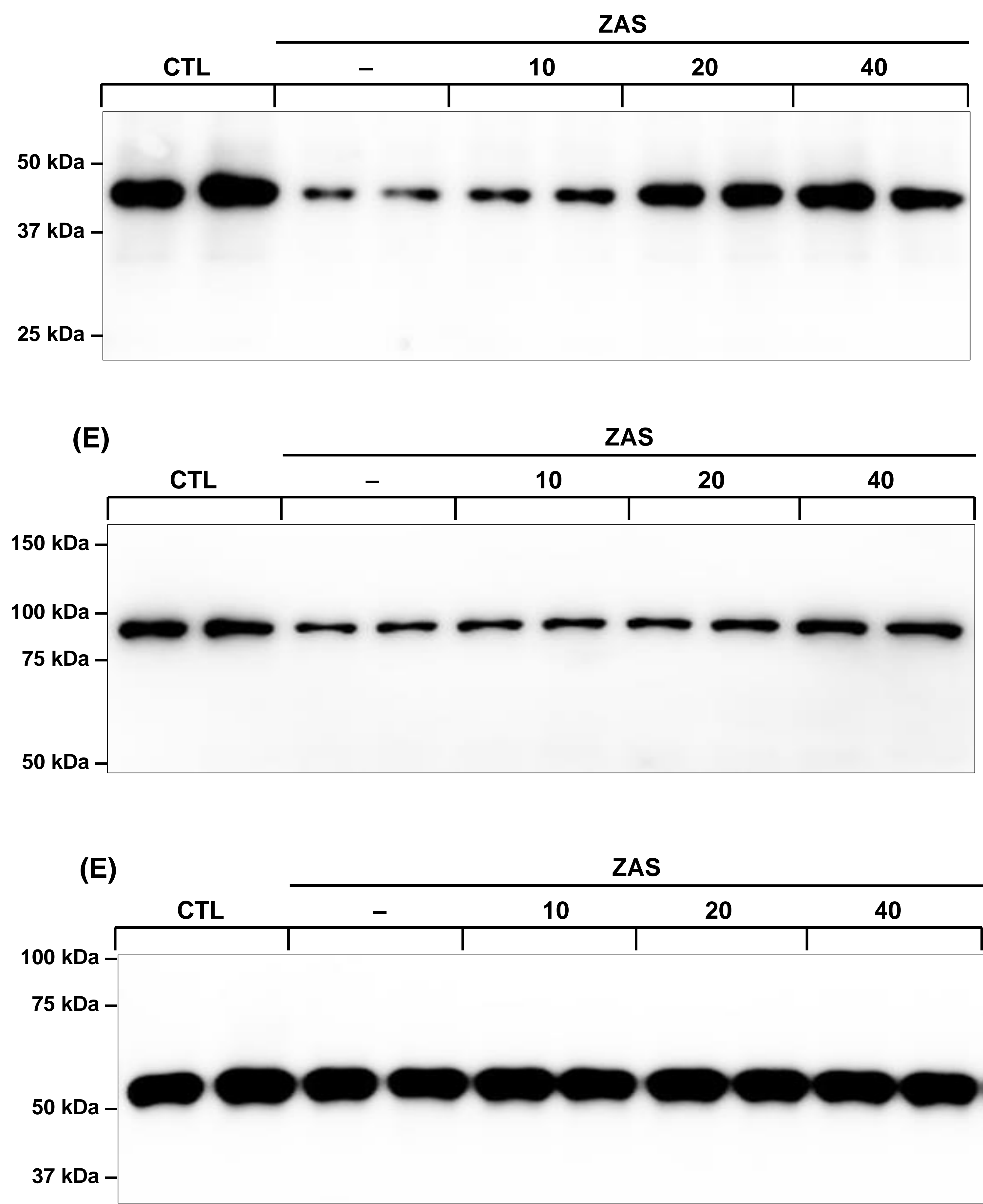

Figure 2E

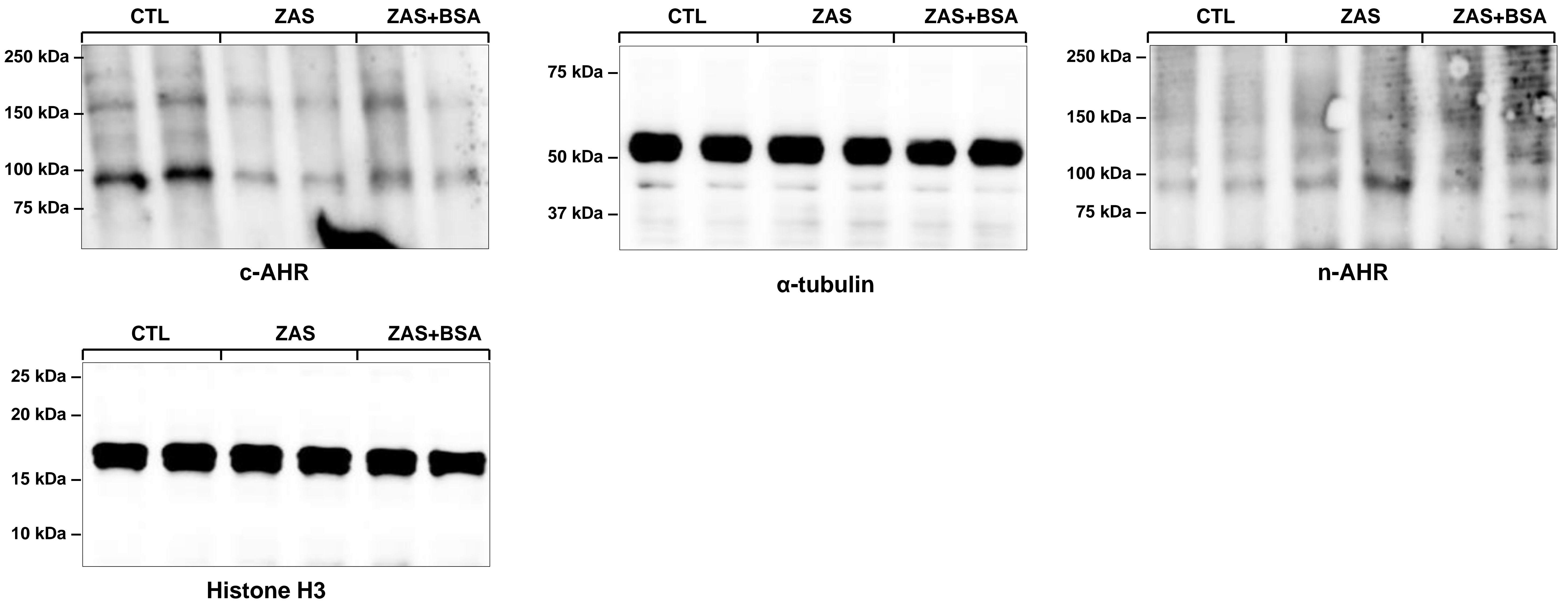

Figure 3B

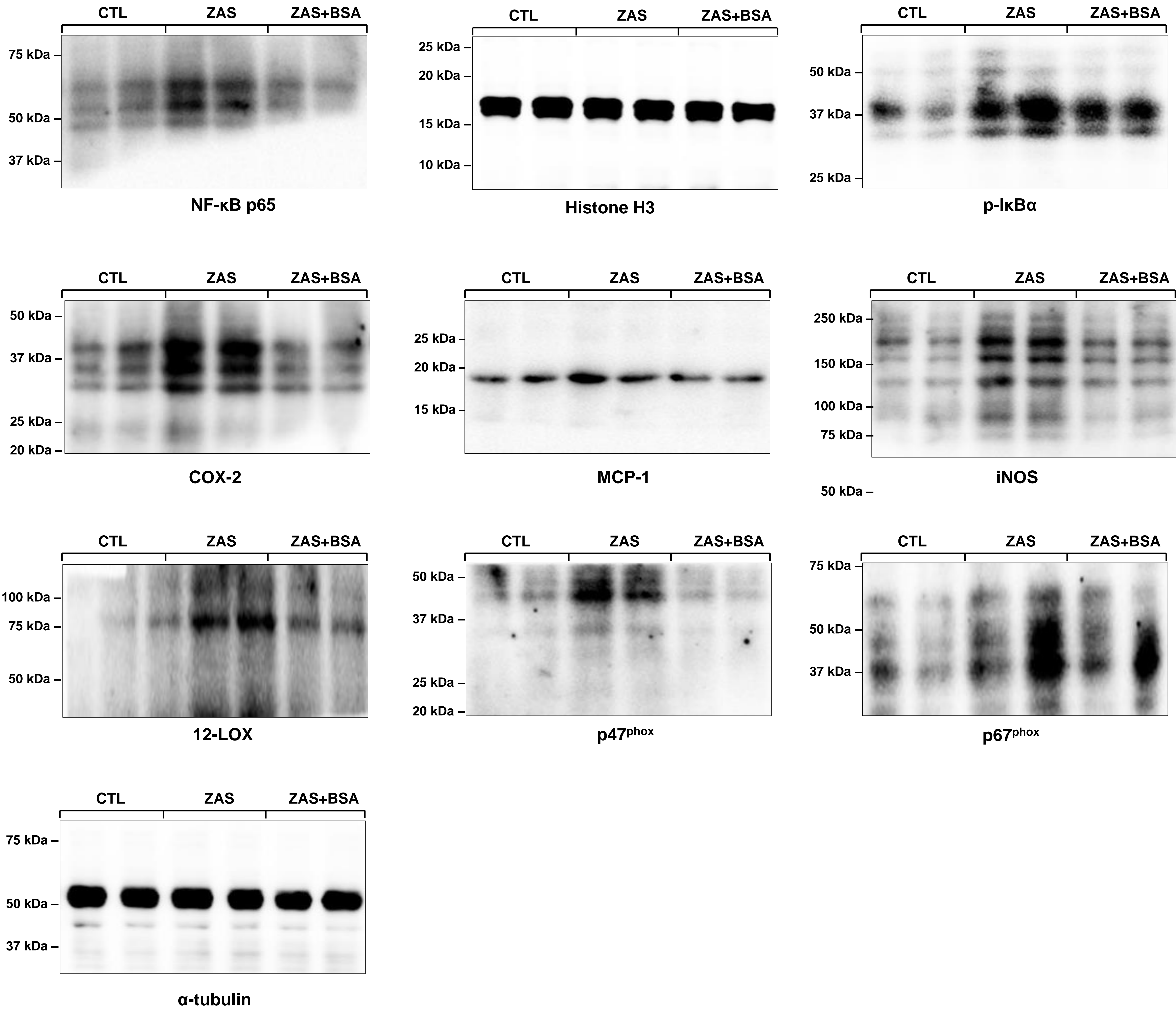

Figure 4B

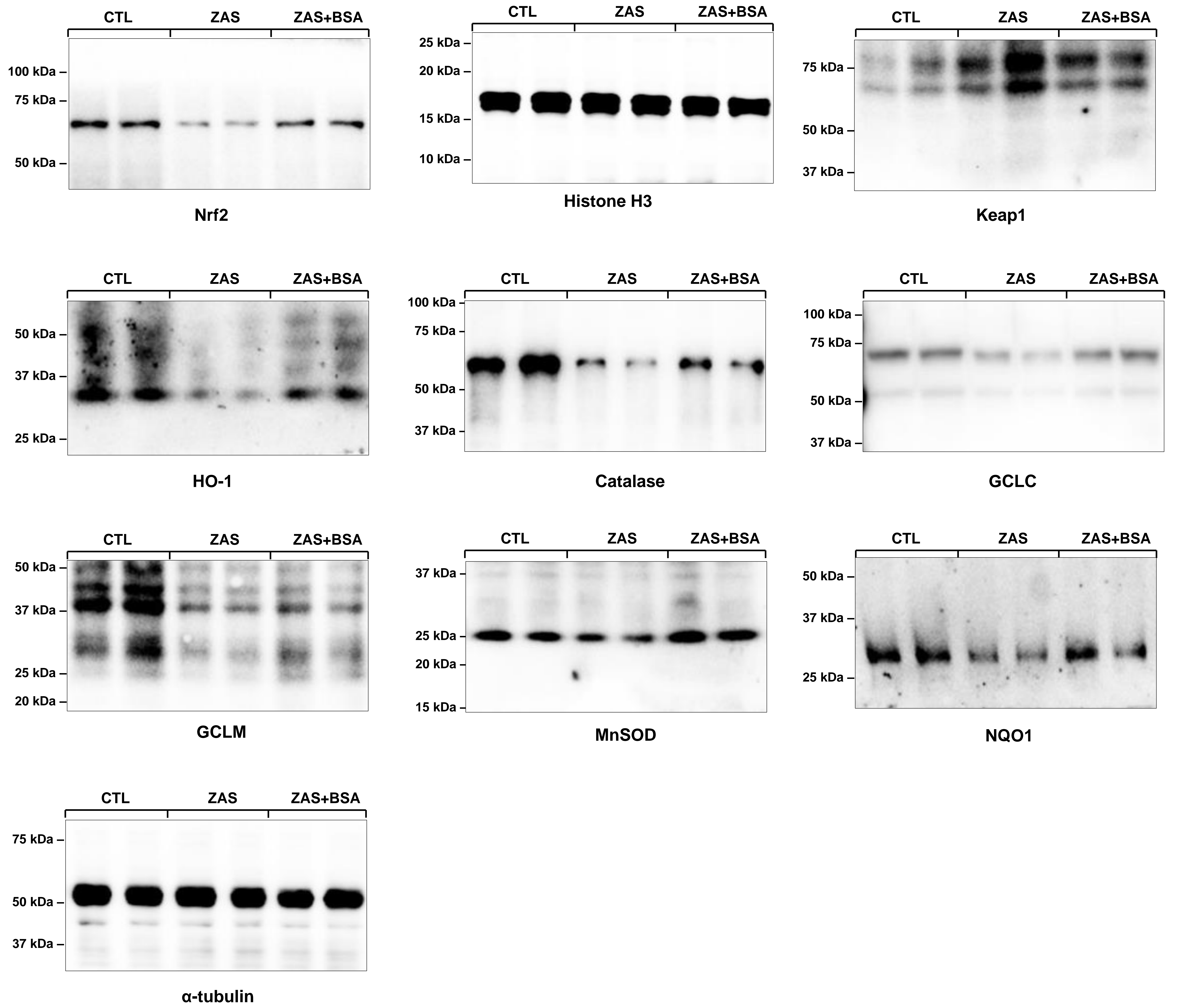

Figure 5A

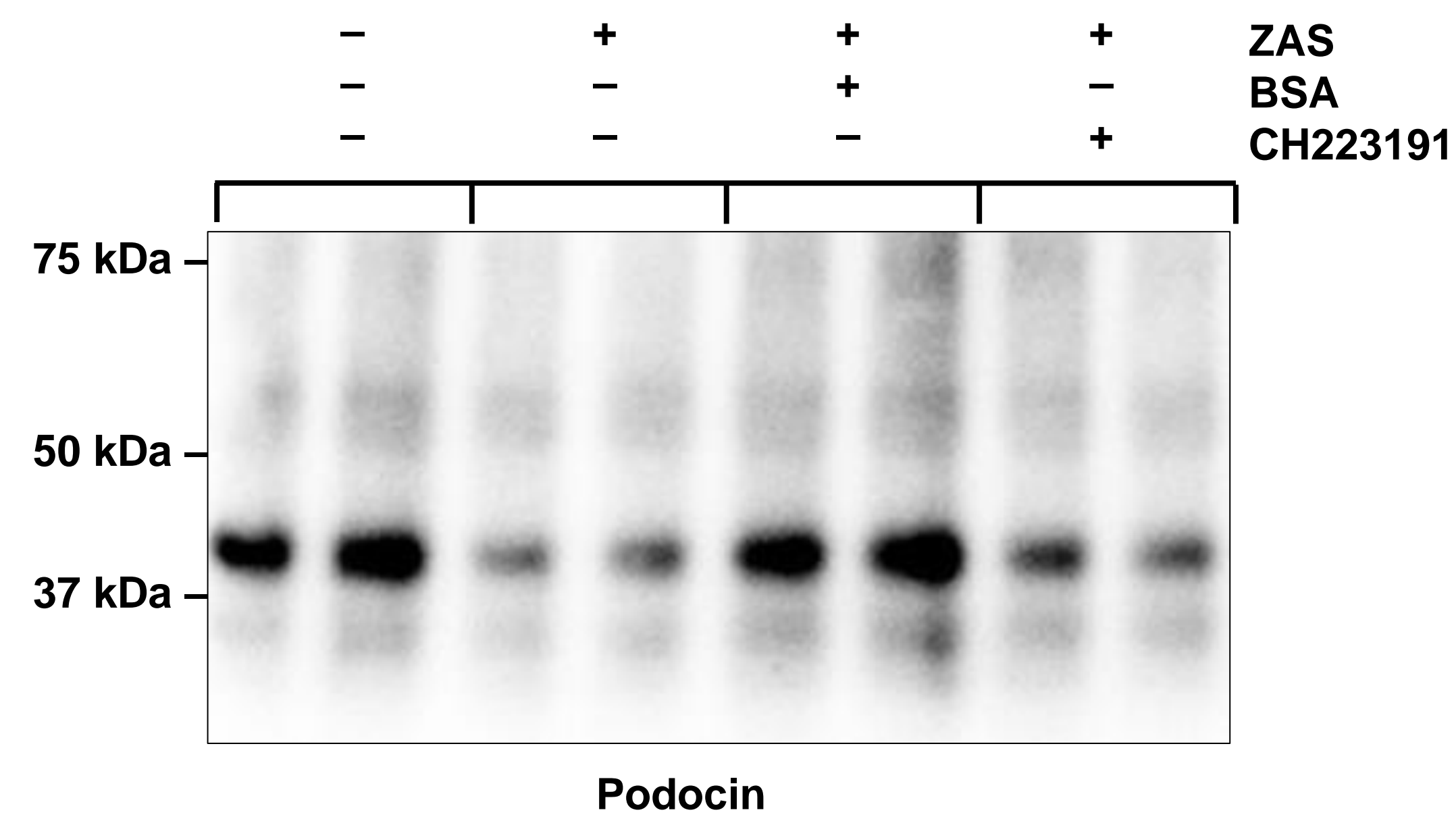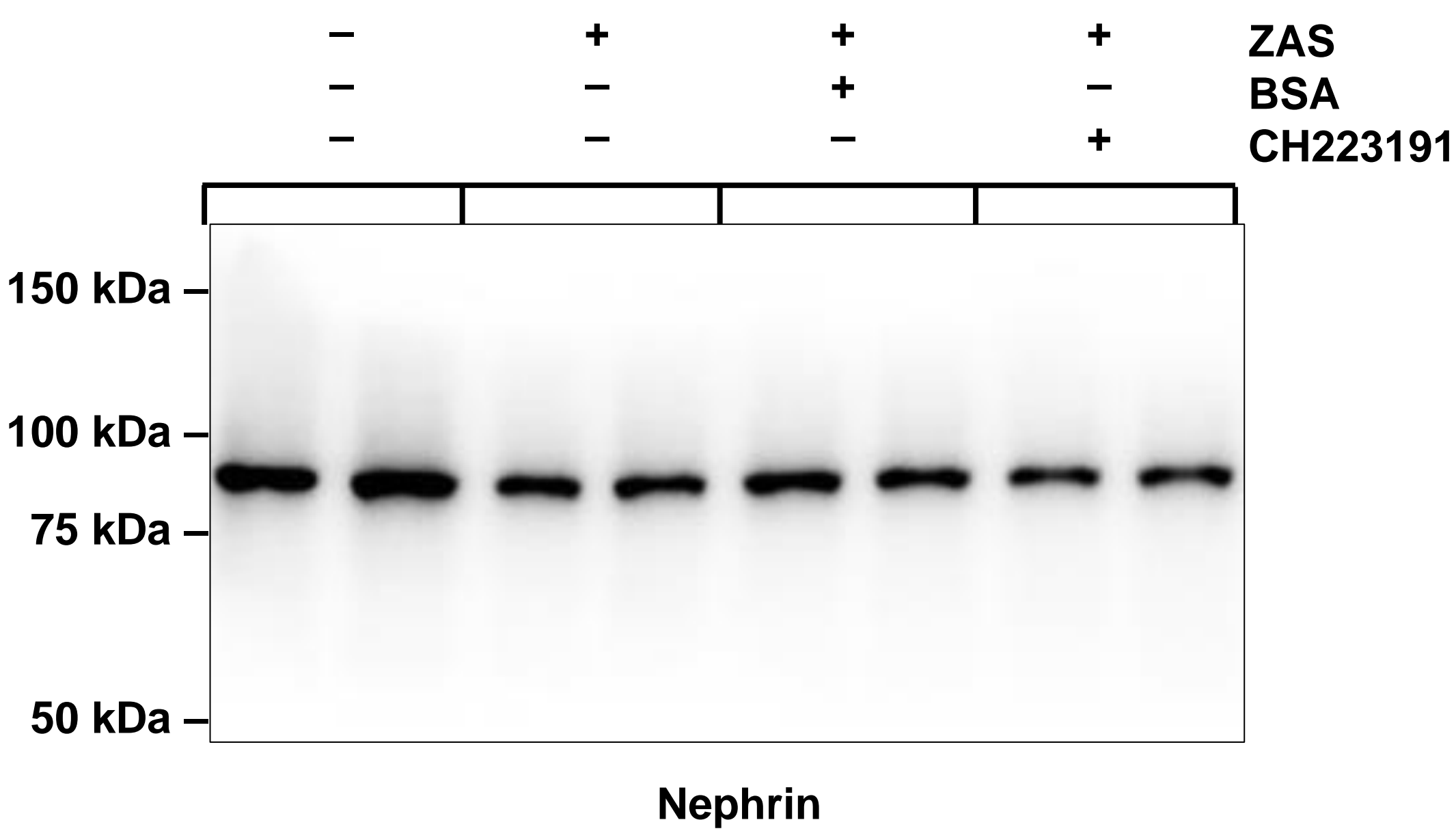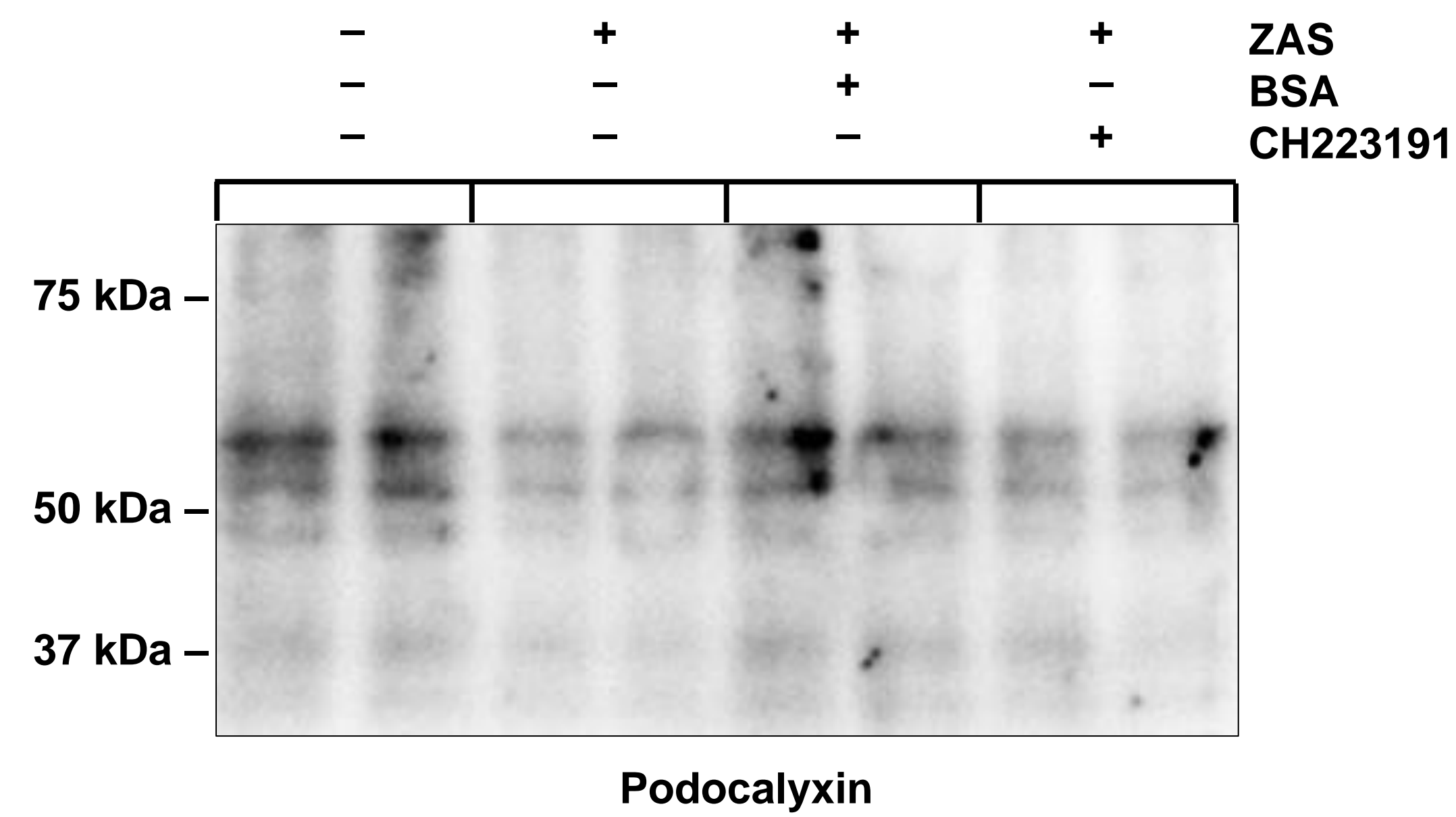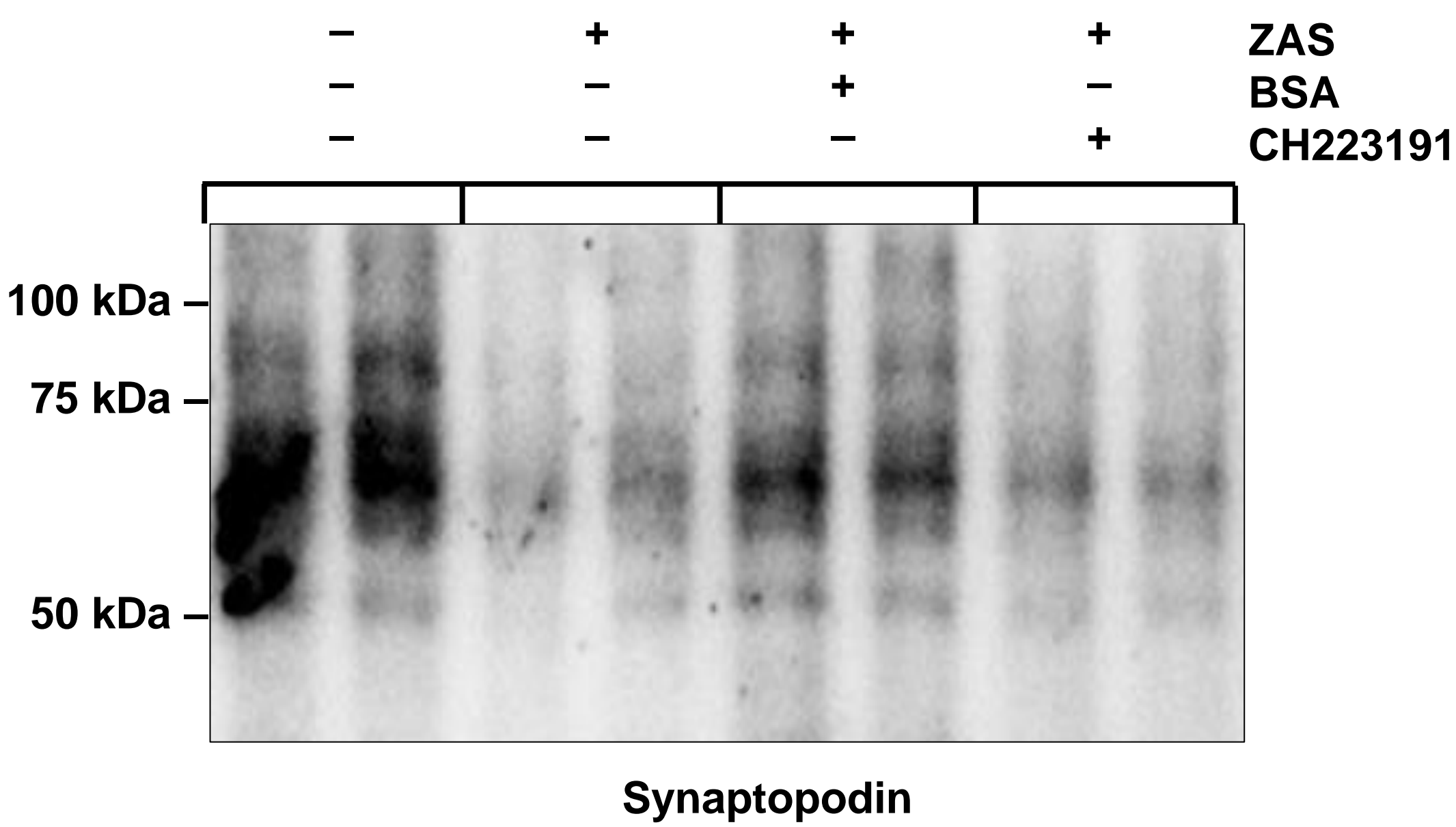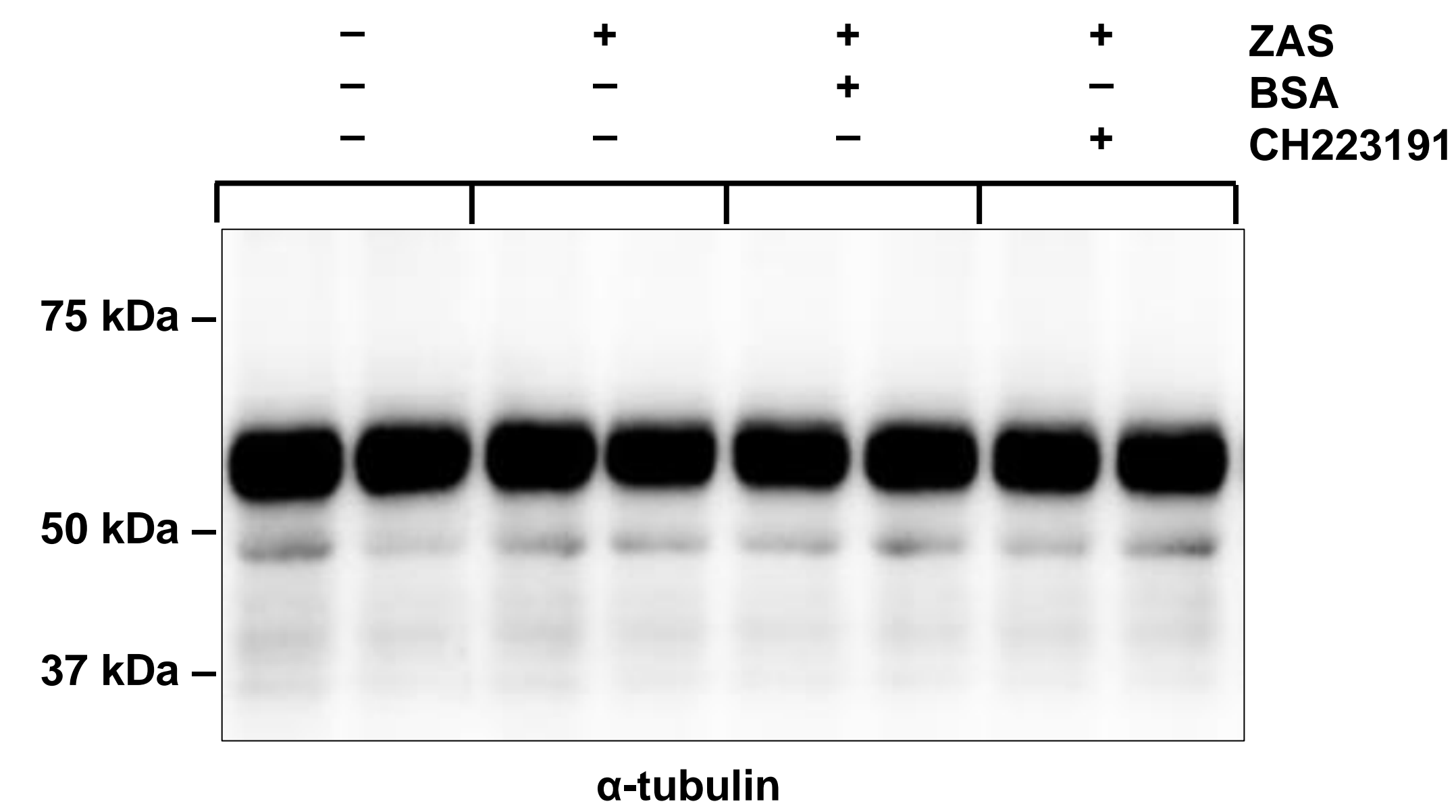

Figure 5C

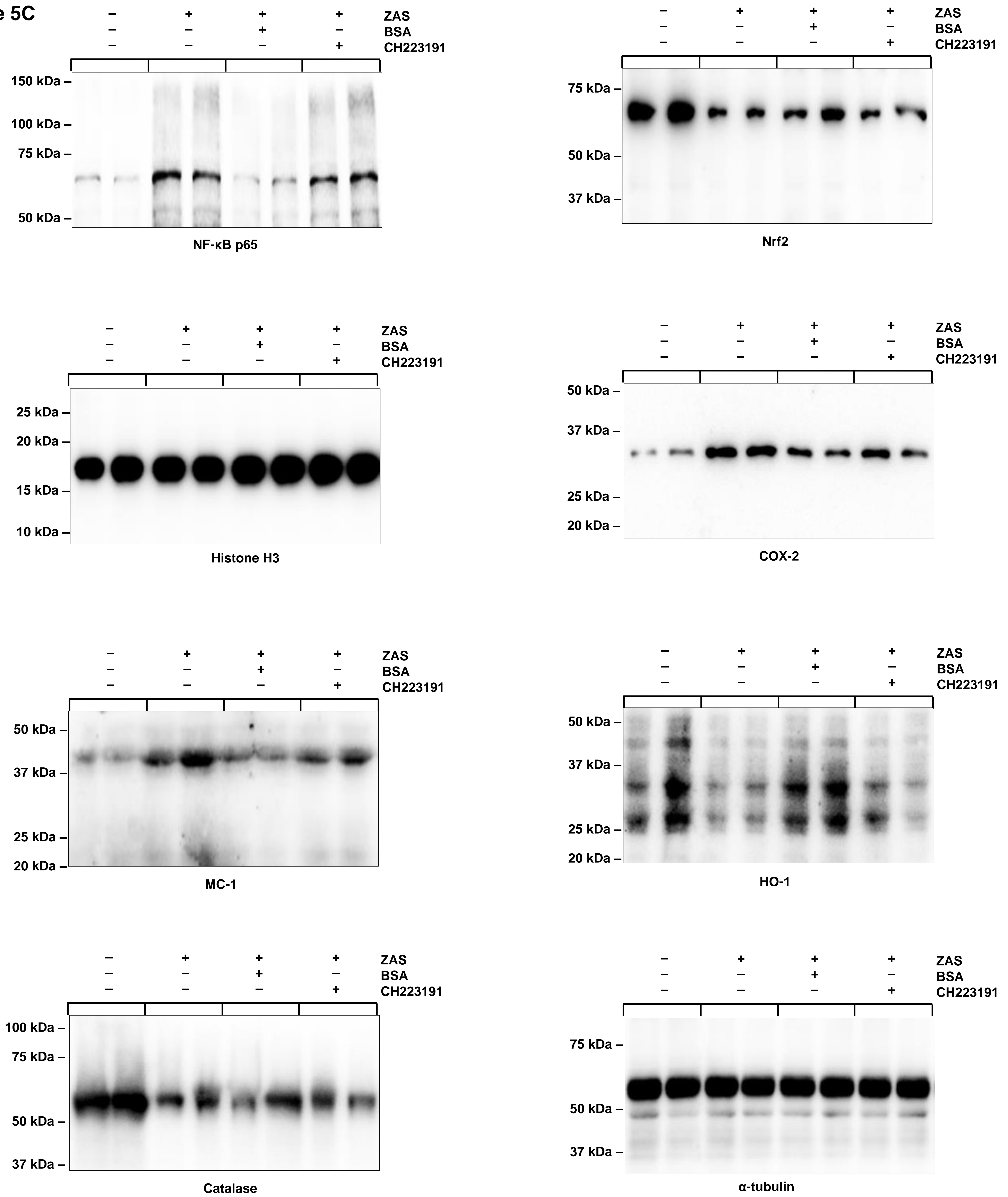

Figure 6A

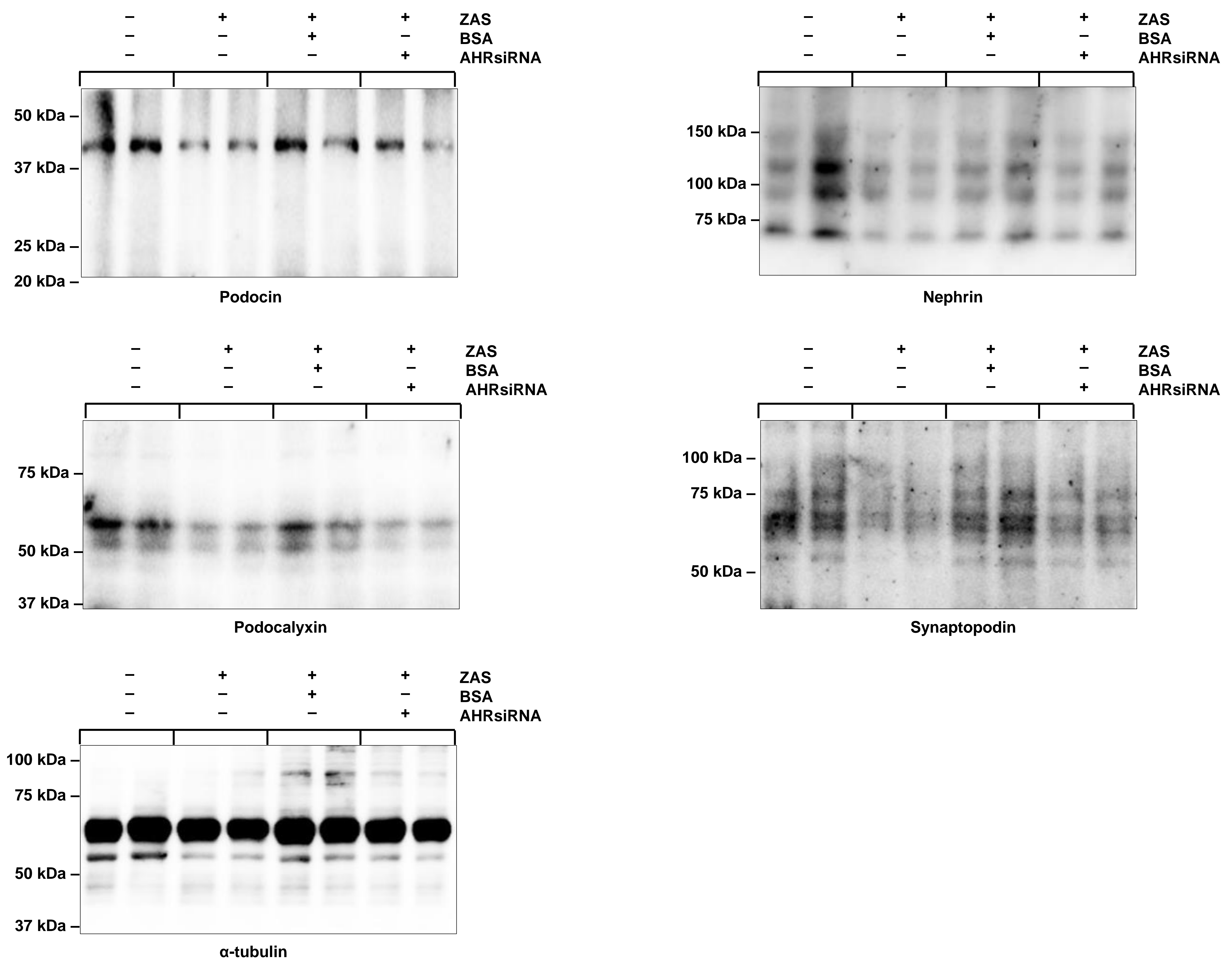

Figure 6C

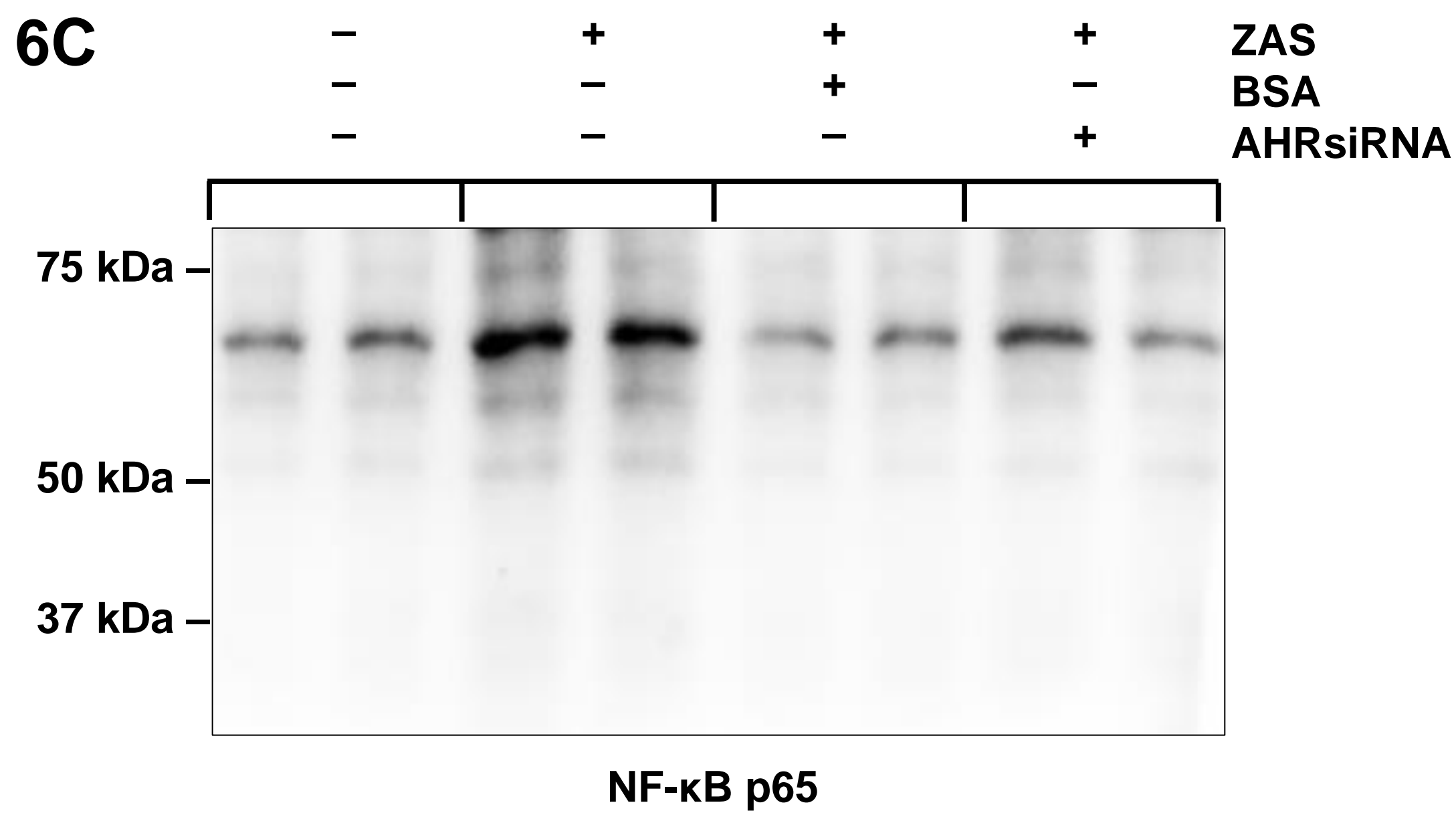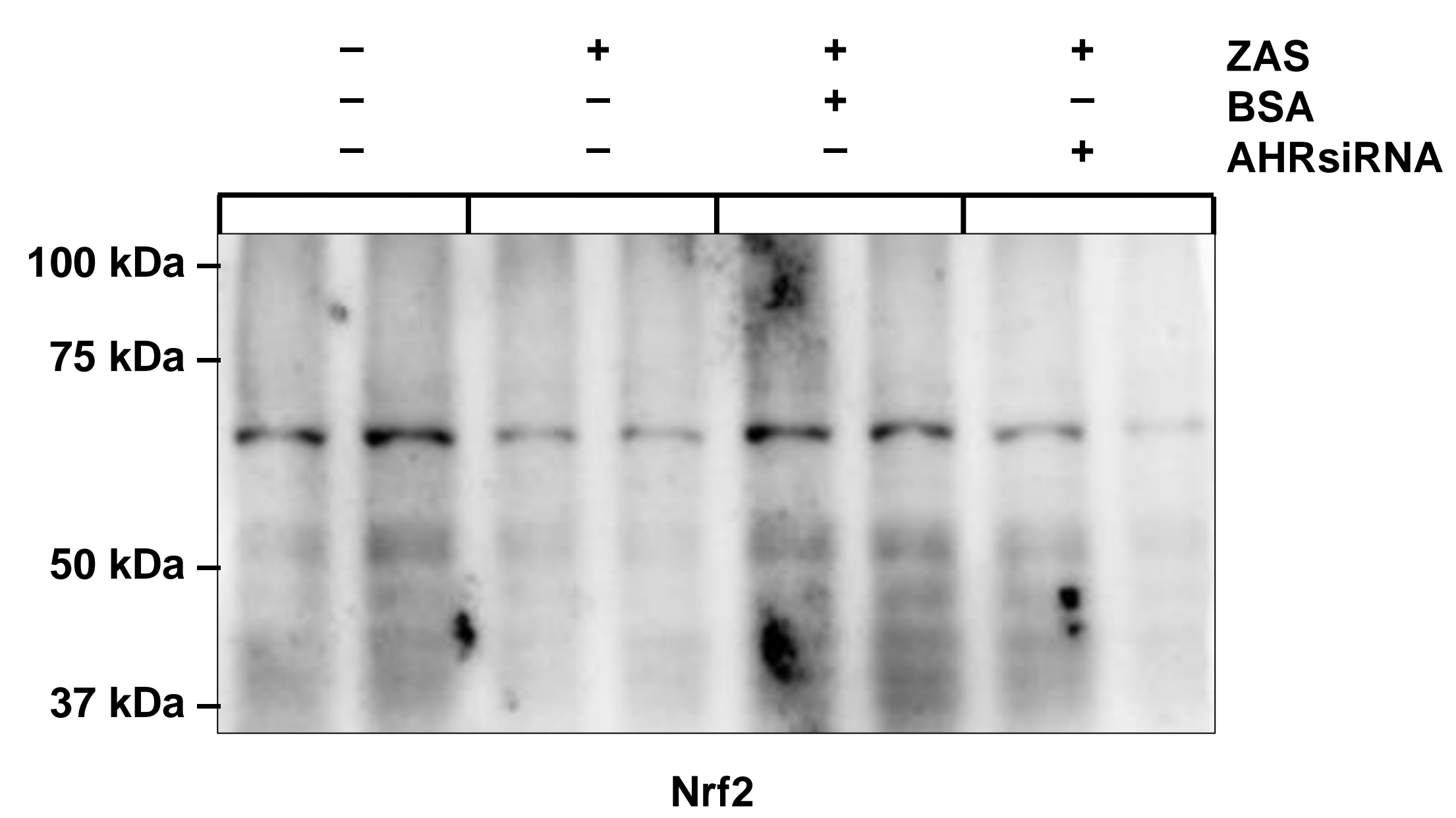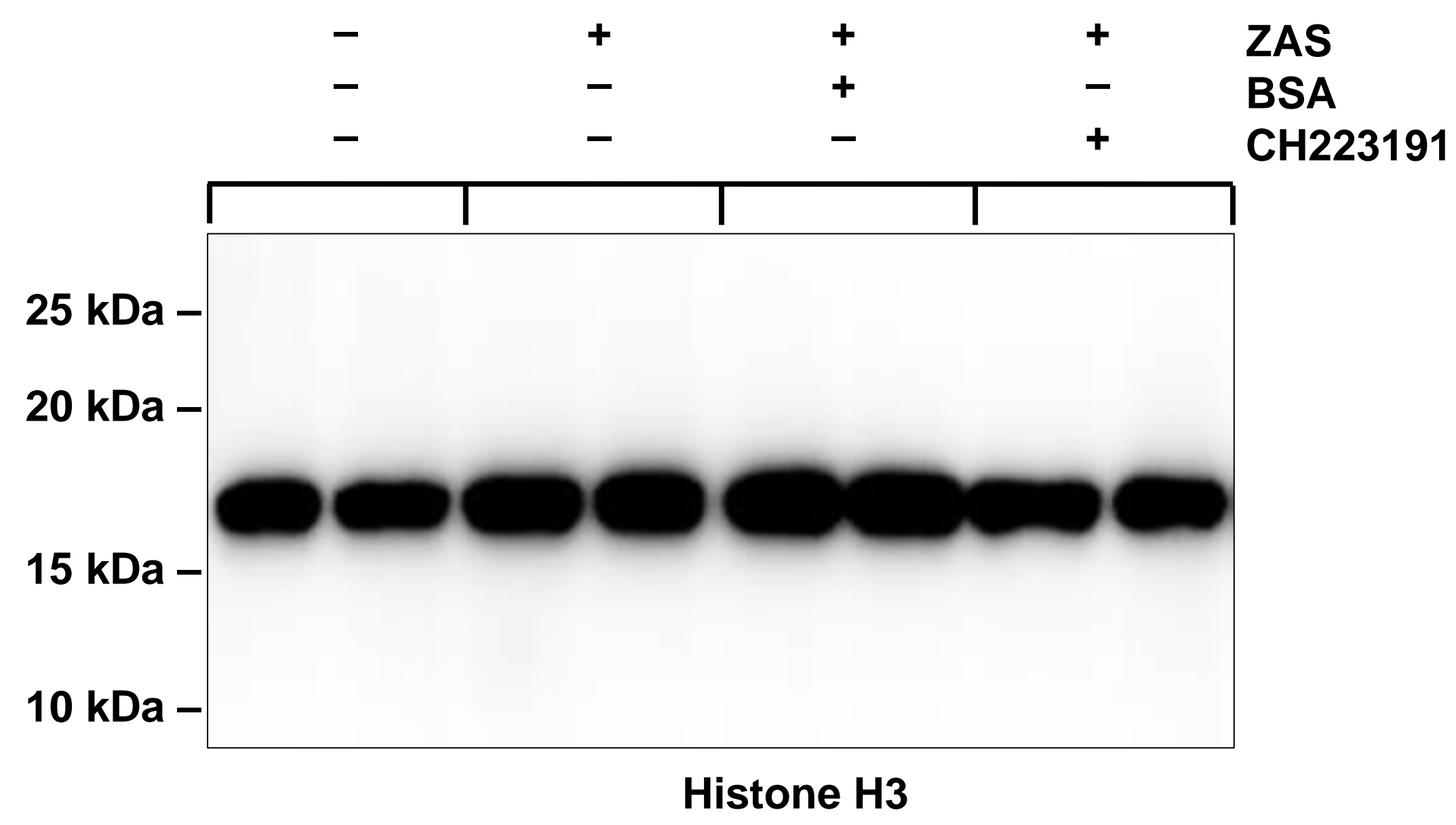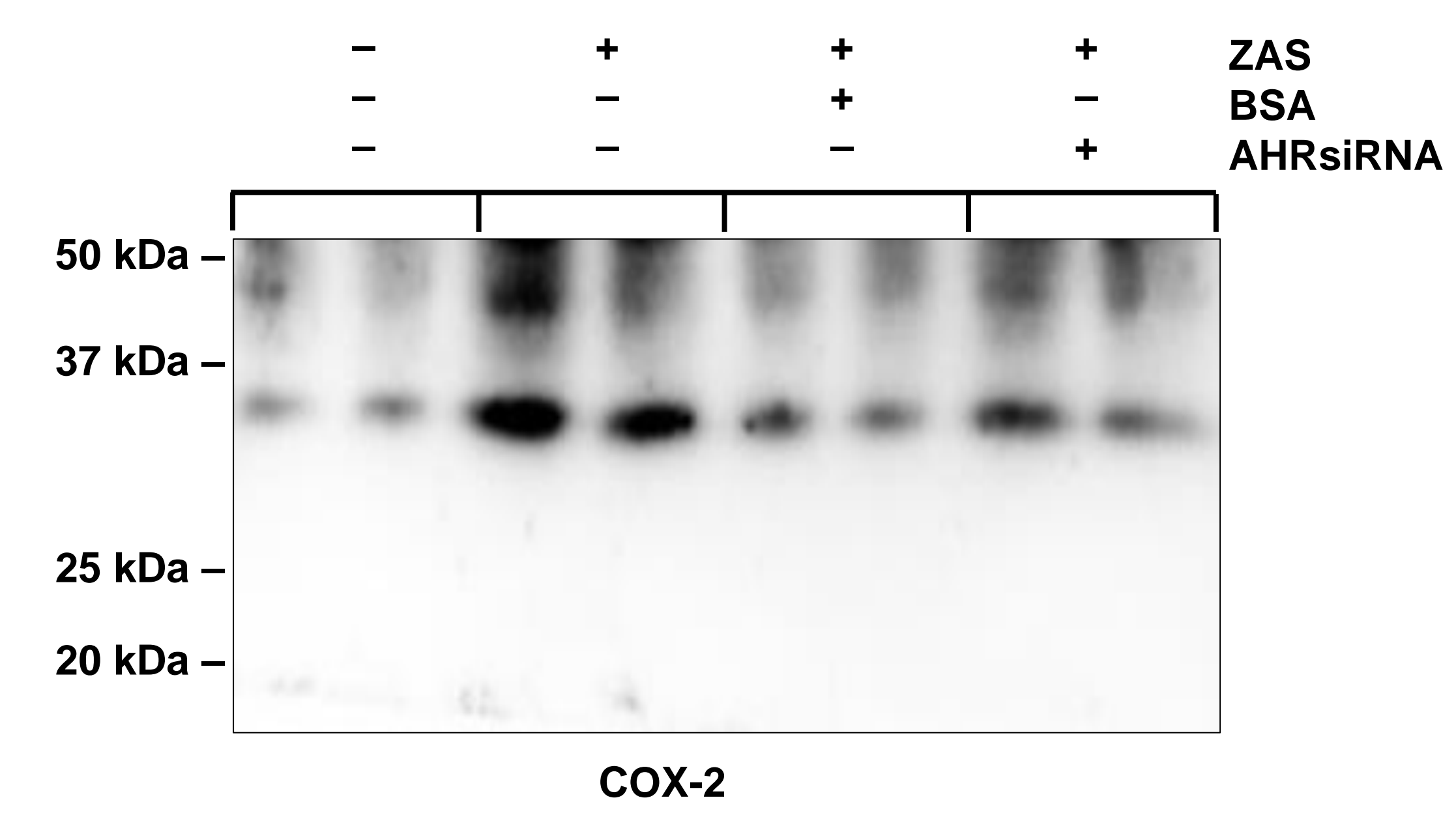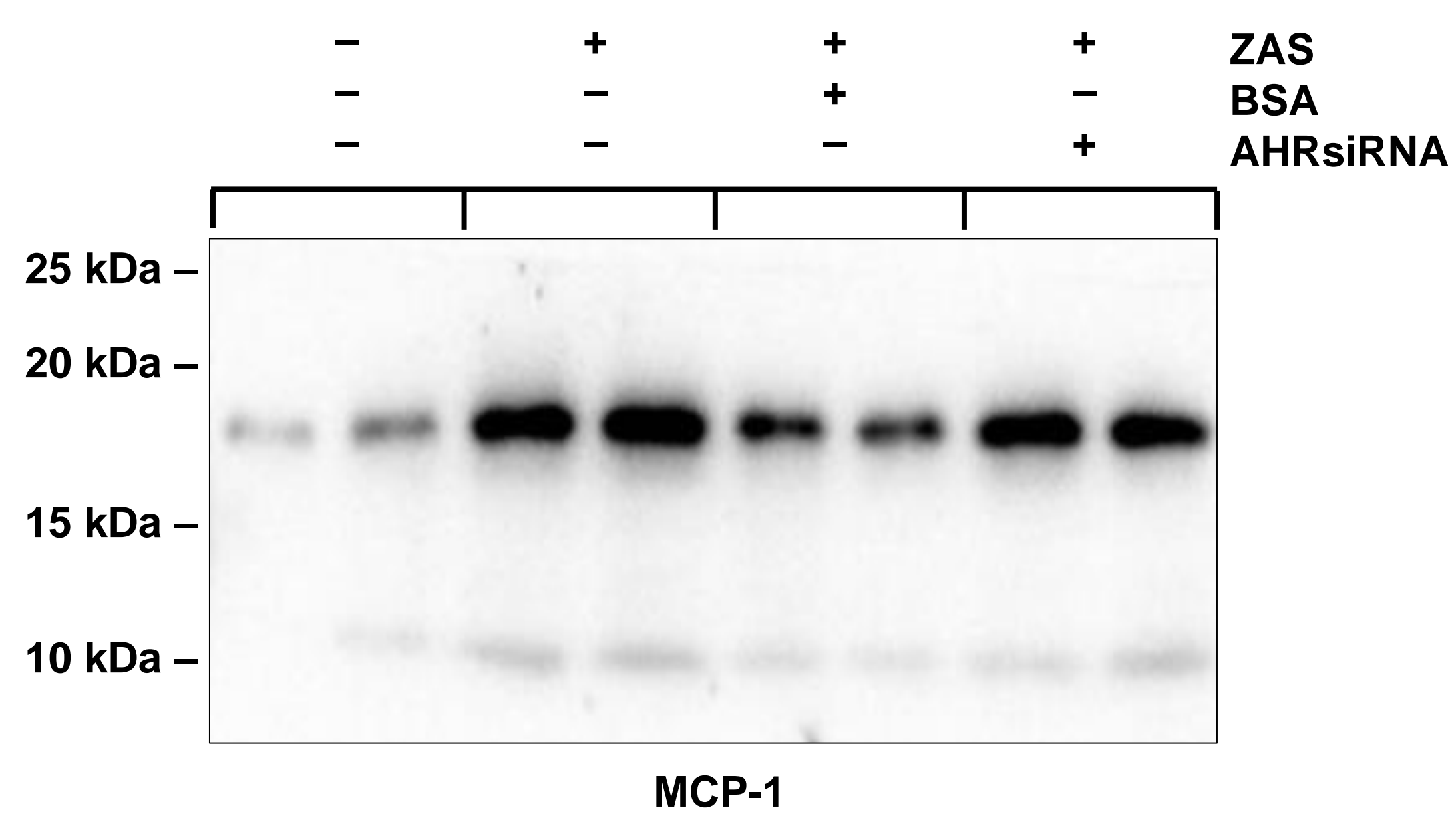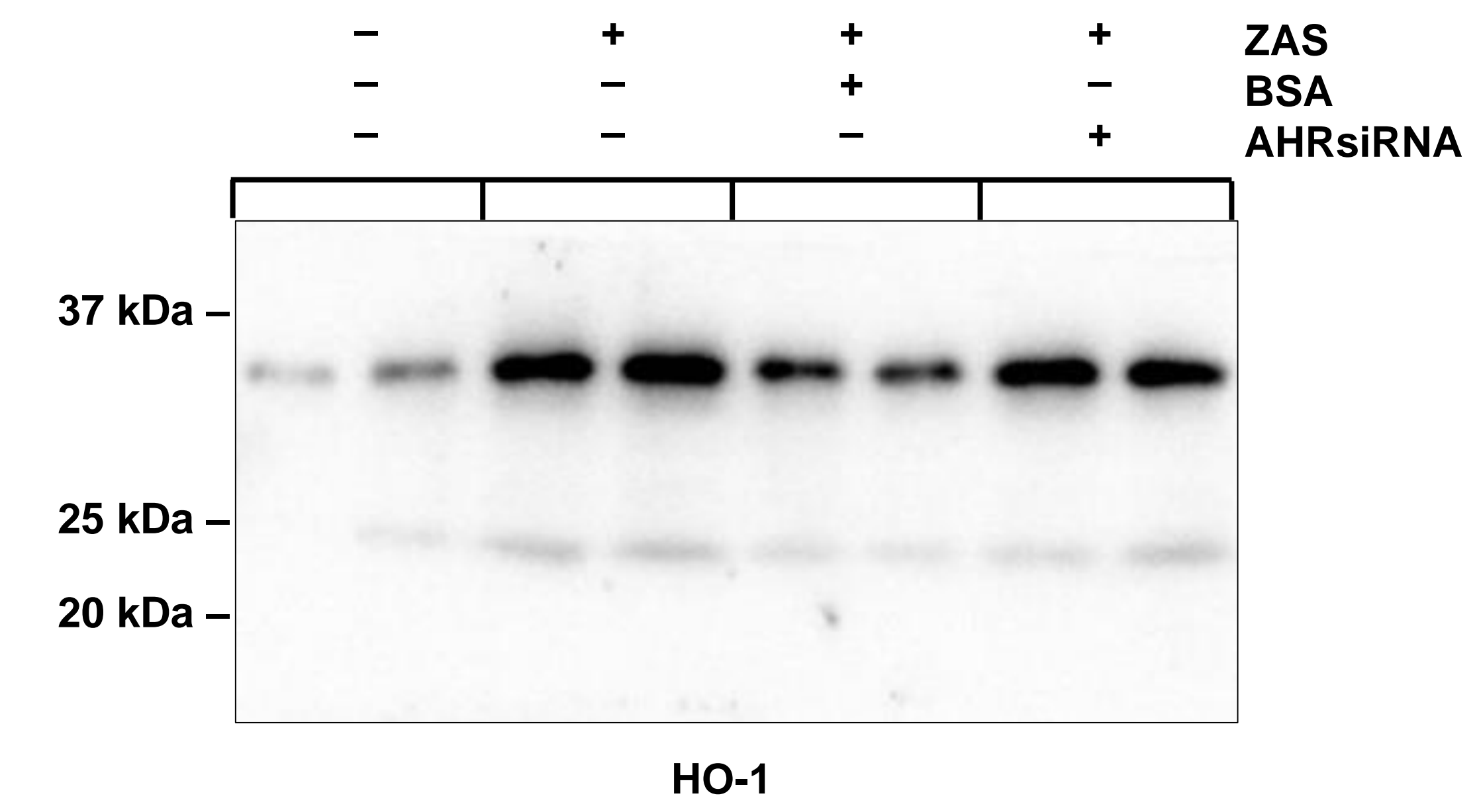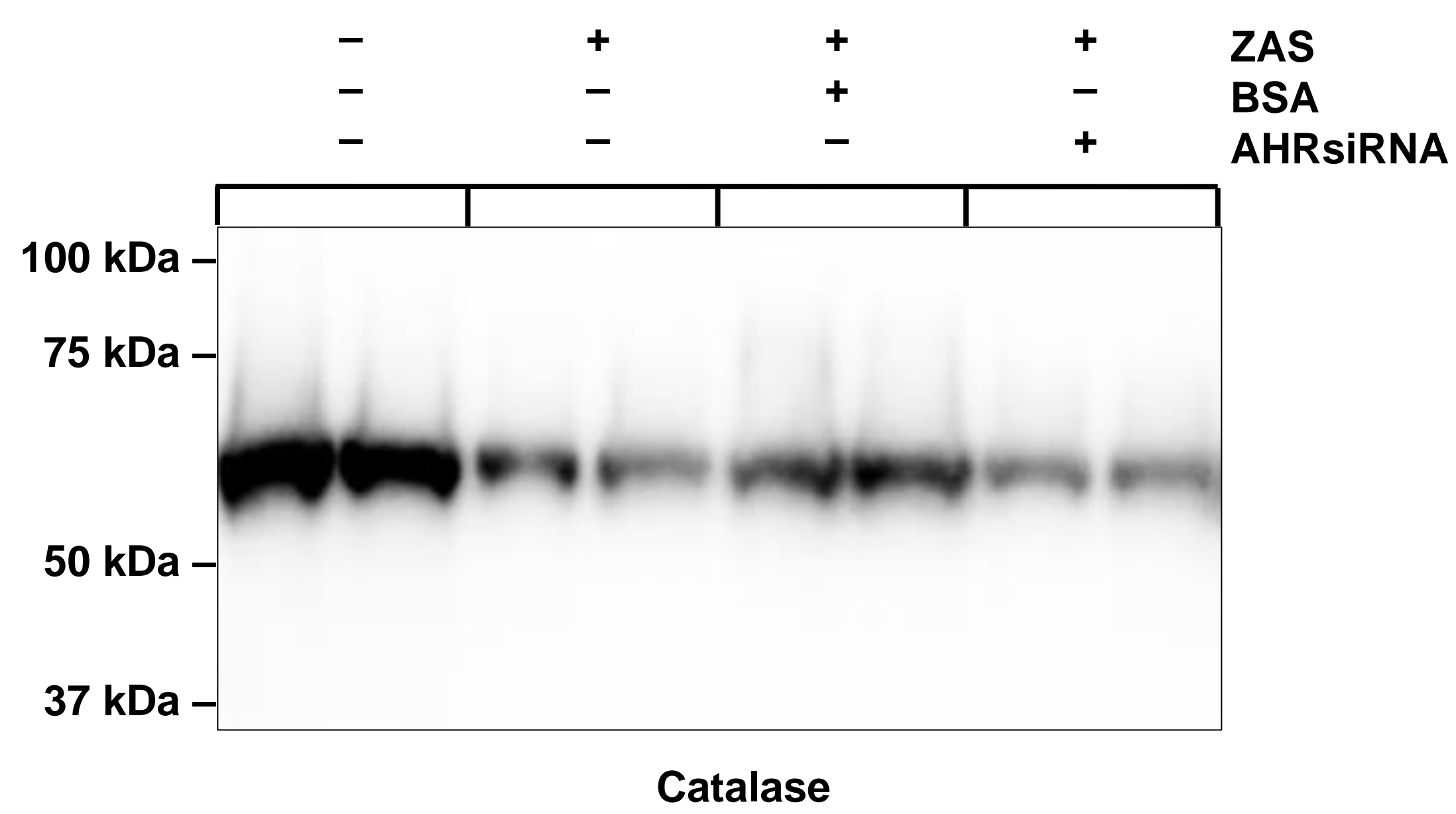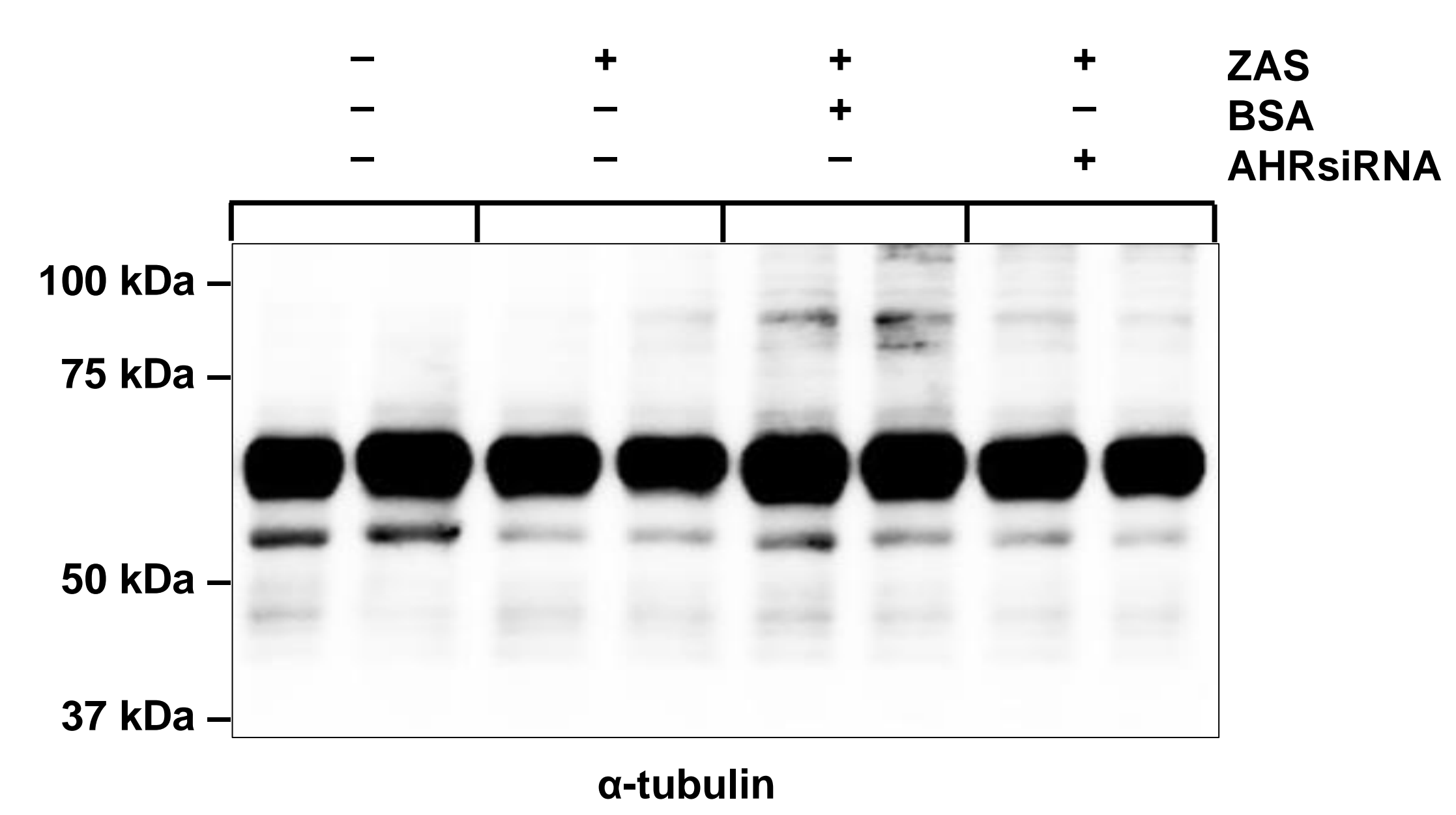

Supplement: Supplementary file 1 [file DataSheet1.PDF]
